# Inflammatory microenvironment regulation and osteogenesis promotion by bone-targeting calcium and magnesium repletion nanoplatform for osteoporosis therapy

**DOI:** 10.1186/s12951-024-02581-7

**Published:** 2024-06-05

**Authors:** Zhenzhen Weng, Jing Ye, Changxiong Cai, Zikang Liu, Yuanyuan Liu, Yingying Xu, Jinghong Yuan, Wei Zhang, Lubing Liu, Junkai Jiang, Xigao Cheng, Xiaolei Wang

**Affiliations:** 1 Department of Orthopaedics, The Second Affiliated Hospital, Jiangxi Medical College, Nanchang University, Nanchang, 330088 Jiangxi P. R. China; 2 School of Chemistry and Chemical Engineering, Nanchang University, Nanchang, 330088 Jiangxi P. R. China; 3 The National Engineering Research Center for Bioengineering Drugs and the Technologies, Institute of Translational Medicine, Nanchang University, Nanchang, 330088 Jiangxi P. R. China

**Keywords:** pH responsiveness, Bone-targeting, Inflammatory microenvironment, Osteogenesis, Osteoporosis therapy

## Abstract

Osteoporosis is the most common bone metabolic disease that affects the health of middle-aged and elderly people, which is hallmarked by imbalanced bone remodeling and a deteriorating immune microenvironment. Magnesium and calcium are pivotal matrix components that participate in the bone formation process, especially in the immune microenvironment regulation and bone remodeling stages. Nevertheless, how to potently deliver magnesium and calcium to bone tissue remains a challenge. Here, we have constructed a multifunctional nanoplatform composed of calcium-based upconversion nanoparticles and magnesium organic frameworks (CM-NH_2_-PAA-Ald, denoted as CMPA), which features bone-targeting and pH-responsive properties, effectively regulating the inflammatory microenvironment and promoting the coordination of osteogenic functions for treating osteoporosis. The nanoplatform can efficaciously target bone tissue and gradually degrade in response to the acidic microenvironment of osteoporosis to release magnesium and calcium ions. This study validates that CMPA possessing favorable biocompatibility can suppress inflammation and facilitate osteogenesis to treat osteoporosis. Importantly, high-throughput sequencing results demonstrate that the nanoplatform exerts a good inflammatory regulation effect through inhibition of the nuclear factor kappa-B signaling pathway, thereby normalizing the osteoporotic microenvironment. This collaborative therapeutic strategy that focuses on improving bone microenvironment and promoting osteogenesis provides new insight for the treatment of metabolic diseases such as osteoporosis.

**Supplementary Information:**

The online version contains supplementary material available at 10.1186/s12951-024-02581-7.

## Introduction

Osteoporosis is considered one of the most common systemic bone metabolism diseases, which is hallmarked by low bone mass and deteriorated bone tissue microstructure, followed by increased bone fragility and fracture susceptibility [1, 2]. The prevalence of osteoporosis has risen sharply as a consequence of the deepened population aging, which has emerged as a major global healthcare issue [3, 4]. Currently, the conventional treatment options for osteoporosis predominantly enclose antiresorptive drugs and anabolic agents, which remain a single therapy modality that inhibits bone resorption or promotes osteogenesis [5, 6]. Nevertheless, the multiple adverse reactions and low patient adherence derived from long-term administration restrict their curative effect [7]. Notably, the osteoporotic bone microenvironment is characterized by a pathological immune environment with disordered secretion of inflammatory cytokines, while this important factor is often overlooked in traditional pharmacological therapy [8, 9]. Given these limitations, it is necessary to develop a new multimodal synergistic treatment strategy that can not only amend the vicious microenvironment in the osteoporotic bone, but also restore the balance between bone resorption and bone formation for osteoporosis remission.

The emergence of metal-based bioactive nanomaterials provides an efficacious alternative for osteoporosis treatment due to the versatile biofunctions of metal ions [10, 11]. For example, magnesium-based nanomaterials are capable of supplying magnesium ions (Mg^2+^), the fourth most abundant metal cations in the body, to participate in osteoblast proliferation and differentiation, further augmenting bone regeneration [12, 13]. Concomitantly, there is increasing evidence that Mg^2+^ exhibits attractive features in anti-inflammatory, which poses considerable potential in ameliorating the inflammatory microenvironment of osteoporosis [14]. Studies have shown that metal-organic frameworks formed by the coordination of metal ions with organic ligands can gradually release metal ions in response to acid conditions, making them ideal candidates for metal ions storage and delivery [15, 16]. Theoretically, magnesium organic framework (Mg-MOF) can be delivered to bone tissue and gradually release Mg^2+^ in response to the acidic microenvironment of osteoporosis, which has the potential to improve the bone microenvironment and promote bone regeneration [17, 18]. It is worth noting that when osteoporosis occurs, there is already a severe calcium deficiency in the bone tissue, and the anti-osteoporosis effect of only magnesium supplementation is limited [19]. Calcium-based nanomaterials can be used as calcium sources in supplements to boost osteogenesis, making them a promising choice for addressing age-dependent calcium deficiency in the osteoporotic microenvironment [20]. Among them, calcium fluoride is one of the luminescent substrates of upconversion nanoparticles, which has been paid extensive attention in the fields of diagnosis and treatment [21, 22]. Moreover, as an essential mineral component in bones, calcium ions (Ca^2+^) can form hydroxyapatite under the control of osteoblasts, while fluoride ions are capable of accelerating the formation of inorganic crystals to increase bone density [23]. Capitalizing on this, the construction of an acid-responsive nanoplatform that simultaneously realizes calcium and magnesium repletion based on calcium fluoride-based nanomaterials and Mg-MOF, which is conducive to the multifunctional cooperative treatment of osteoporosis.

Conventional methods of supplementing calcium and magnesium always have low bioavailability and many side effects [24]. Thus, it is required to deliver highly bioactive calcium and magnesium into bone tissue in a targeted manner that will benefit osteoporosis alleviation by taking advantage of the plasticity of metal ions. Bisphosphonates can be preferentially deposited in bone tissue and modified on the surface of nanomaterials to achieve bone-targeted delivery of materials, thereby completing the *in-situ* supplementation of calcium and magnesium in bone tissue of osteoporosis [25]. After the targeted delivery of the material to the lesion, the responsive and sustained release of pivotal metal ions can be implemented by utilizing the distinguishing feature of the highly enriched acidic microenvironment in osteoporosis, which is crucial for anti-osteoporosis [26, 27].

**Scheme 1:**
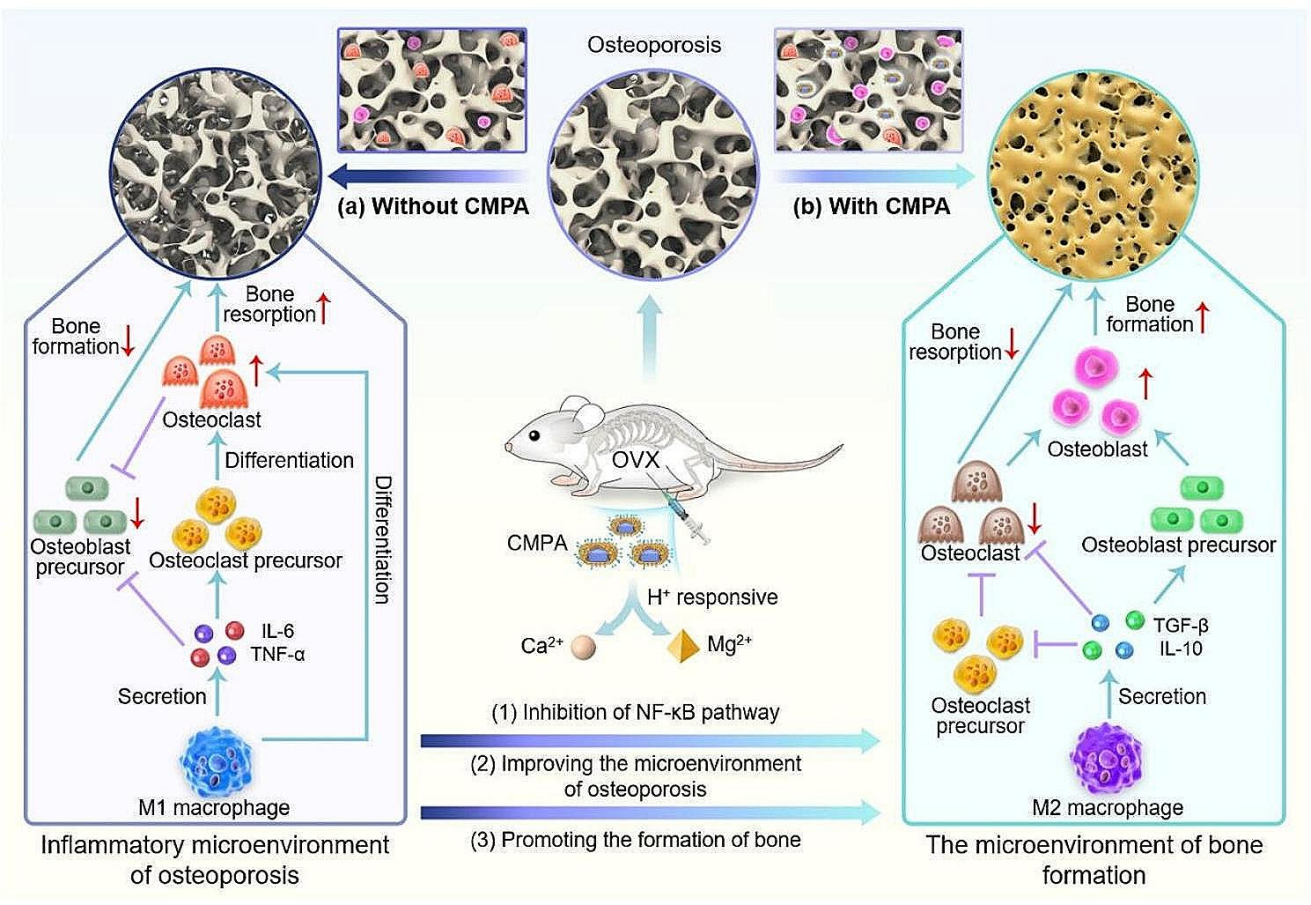
Schematic illustration of the application of CMPA for osteoporosis therapy. (**a**) The inflammatory microenvironment in osteoporosis can lead to the formation of a large number of osteoclasts, further aggravating the deterioration of osteoporosis. (**b**) In the therapy of ovariectomy (OVX)-induced mice, CMPA could release Mg^2+^ and Ca^2+^ in response to acidic microenvironment, which was beneficial for improving the microenvironment of osteoporosis and promoting the formation of bone

Herein, a nano-treatment platform with bone-targeting and pH-responsive properties was customized for osteoporosis therapy, which consisted of calcium-based upconversion nanoparticles (designated as CaF_2_) and Mg-MOF (CM-NH_2_-PAA-Ald, denoted as CMPA), providing *in-situ* repletion of calcium and magnesium in osteoporotic bone tissue (Scheme 1). On the one hand, the core structure CaF_2_ was encapsulated by Mg-MOF as a shell structure, which was capable of avoiding the direct binding of alendronate sodium (Ald) to CaF_2_, so that CMPA could efficiently accumulate in bone tissue to achieve bone-targeted therapy for increasing efficacy and reducing side effects [25]. On the other hand, the nanoplatform re-established the osteoporosis inflammatory microenvironment and played a coordinating role in promoting bone formation by introducing a variety of bioactive mineral ions, ultimately achieving effective treatment of osteoporosis.

## Materials and methods

### Materials

Calcium chloride hexahydrate (CaCl_2_·6H_2_O), 2,5-dihydroxyterephthalic acid (DHTA), N,N-dimethylformamide (DMF), sodium citrate (Cit), ammonium fluoride (NH_4_F), sodium hydroxide (NaOH), alendronate sodium (Ald), tert-butanol and rhodamine B (RhB) were purchased from Macklin Biochemical Co., Ltd. (Shanghai, China). Anhydrous ethanol was bought from Xilong Scientific Co., Ltd. (China). Ytterbium nitrate pentahydrate (Yb(NO_3_)_3_·5H_2_O) and erbium nitrate hexahydrate (Er(NO_3_)_3_·6H_2_O) were obtained from Shandong Desheng New Material Co., Ltd. (Shandong, China). Magnesium nitrate hexahydrate (Mg(NO_3_)_2_·6H_2_O) was bought from Sinopharm Chemical Reagent Co., Ltd. (Shanghai, China). Polyacrylic acid (PAA, Mw = 2,000) was procured from Acros. Hydroxyapatite (HAP) was purchased from Sigma-Aldrich (USA). Cell counting kit-8 (CCK-8), penicillin-streptomycin solution, fluorescein 5-isothiocyanate (FITC), phosphate buffer solution (PBS), 2’,7’-dichlorofluorescein diacetate (DCFH-DA), tartrate resistant acid phosphatase (TRAP) stain kit and alkaline phosphatase (ALP) staining kit were obtained from Solarbio Technology Co., Ltd. (Beijing, China). Alizarin red S (ARS) staining kit, Calcein-acetoxymethyl ester (Calcein-AM) and propidium iodide (PI) were bought from Bestbio Biotech Co., Ltd. (Shanghai, China). Fetal bovine serum (FBS) and Dulbecco’s modified eagle medium (DMEM, glucose concentration: 4.5 g L^− 1^) were purchased from HyClone (USA). Enzyme-linked immunosorbent assay (ELISA) kits were bought from Camilo Biological Co., Ltd. (Nanjing, China). TRIzol reagent was obtained from Beyotime Biotechnology Co., Ltd. (Shanghai, China). Mouse macrophages RAW264.7, human umbilical vein endothelial cells (HUVECs), human bone marrow mesenchymal stem cells (hMSCs) and mouse pre-osteoblast cells (MC3T3-E1) were supplied by the Second Affiliated Hospital of Nanchang University (Nanchang, China).

### Characterizations

The morphology and element distribution of samples were examined by transmission electron microscopy (TEM, Thermo/Tecnai G2 20, United States) and scanning electron microscope (SEM, Zeiss/Sigma300, Japan). The structure of materials was characterized by X-ray diffraction (XRD, Bruker, D8 ADVANCE, Germany) and X-ray photoelectron spectroscopy (XPS, Thermo Fisher, ESCALAB250Xi, United States). Ultraviolet-visible (UV-vis) spectra and luminescence spectra were collected on a UV-2600 spectrophotometer (Shimadzu, Japan) and fluorescence spectrophotometer (FL 970, Techcomp). Fourier transform infrared spectroscopy (FTIR) analysis was performed on a FTIR spectrometer (Nicolet, Nicolet 5700, USA). The zeta potentials of the materials were monitored using a Zetasizer Ultra (Malvern, UK). The elements contained in the materials were determined by inductively coupled plasma (ICP) emission spectrometer (VARIAN715-ES, USA).

### Synthesis of calcium fluoride upconversion nanoparticle

The calcium fluoride upconversion nanoparticles were prepared *via* the hydrothermal method reported previously with some modifications [22]. Initially, we synthesized the CaF_2_:Yb/Er upconversion nanoparticles. Analytical grade CaCl_2_·6H_2_O, Yb(NO_3_)_3_·5H_2_O and Er(NO_3_)_3_·6H_2_O were dissolved in 10 mL deionized water to obtain a mixed solution of 10 mmol according to a molar ratio of Ca: Yb: Er = 78%: 20%: 2%. After adding 20 mmol of Cit aqueous solution (40 mL) for 30 min, 5 mmol of NH_4_F (30 mL) was slowly added under intense stirring for another 30 min. The above solution was sealed in a Teflon-lined autoclave and kept at 190℃ for 6 h, followed by centrifugation at 10,000 rpm for 30 min and washing thrice with deionized water. Finally, the collected CaF_2_:Yb/Er upconversion nanoparticles (denoted as CaF_2_) were dispersed in 5 mL of deionized water for later use.

### Preparation of CaF_2_@Mg-MOF (CM) nanoparticle

In a typical procedure, 3 mL CaF_2_ was re-dispersed in the solution of 45 mL DMF and 3 mL ethanol. After that, 2.25 mmol of Mg(NO_3_)_2_·6H_2_O was introduced and sonicated for 30 min, followed by adding 1.9 mmol of DHTA with ultrasound for another 30 min. The mixture was then placed in a Teflon-lined autoclave and heated continuously at 125℃. After 5 h of reaction, the CM nanoparticles were collected and purified with DMF and ethanol alternately by repeated centrifugation at 11,000 rpm for 20 min.

### Fabrication of amino-functionalized CM (CM-NH_2_) nanoparticle

For amino functionalization, the obtained CM nanoparticles were dissolved in 10 mL ethanol and mixed with 0.5 mmol of prepared ethylenediamine. Subsequently, the mixture was refluxed for 2 h under the protection of argon. After cooling, the resulting nanomaterial (CM-NH_2_) was washed with ethanol three times and dispersed in ethanol.

### Synthesis of bone-targeted CM nanoparticle

First of all, Ald was anchored on PAA to form a bone-targeting ligand (PAA-Ald) according to our previous work [28]. Afterward, PAA-Ald was coated on the surface of CM-NH_2_ through electrostatic adsorption to prepare CM-NH_2_-PAA-Ald (CMPA). Specifically, the above CM-NH_2_ solution was diluted with deionized water at a volume ratio of 20: 11, and then 500 µL PAA-Ald (0.1 g mL^− 1^) was added slowly dropwise to form a transparent solution, which was further stirred continuously for 0.5 h before centrifugation (11,000 rpm, 30 min) to obtain CMPA. Ultimately, CMPA nanoparticles were washed once with water and twice with ethanol, which were then dried under vacuum.

### Assessment of bone-targeting ability in vitro and in vivo

To evaluate the targeting capacity of the nanoparticles, 1 mg mL^− 1^ of CM or CMPA was mixed with 10 mg HAP in 10 mL deionized water, followed by stirring for varying times (10 min, 30 min, 1 h, 3 h and 6 h). Subsequently, the supernatant and precipitate at diverse time points were collected through centrifugation, in which the supernatant was used for the determination of the fluorescence spectrum, while the precipitate was characterized by SEM after washing three times with deionized water and drying. For the in vivo bone-targeting ability test, FITC-labeled CMPA (1 mg mL^− 1^) was suspended in sterile saline for use. All experimental procedures were conducted in accordance with the institutional guidelines for animal care and approved by the animal ethics committee of Nanchang University (Nanchang, China, NCULAE-20221228021). After anesthesia with isoflurane, FITC-labeled CMPA was injected intraperitoneally into 10-week-old female Kunming (KM) mice (Hunan SJA Laboratory Animal Co., Ltd., Changsha, China). Thereafter, mice were euthanized by inhalation of isoflurane at various time intervals to harvest bone tissue, and the fluorescence intensity was recorded using an in vivo imaging system (IVIS, PerkinElmer).

### The pH responsiveness of CMPA

5 mg CMPA was thoroughly mixed with 20 mL PBS with different pH values (4.5, 5.5, 6.5 and 7.5). After 1 day, the appropriate amount of supernatant was diluted, and the characteristic absorption peak of the released DHTA near 240 nm was measured by a UV-vis spectrophotometer. Meanwhile, the solutions of the above groups were collected and dropped on the silicon wafer for sample preparation, and the morphological changes of CMPA were further observed by SEM. In addition, an ICP emission spectrometer was adopted to detect the content of Mg^2+^ and Ca^2+^ released by CMPA in PBS with different pH values.

### Anti-osteoclast efficiency in vitro

RAW264.7 cells were plated at a density of 2 × 10^3^ cells per well in 96-well plates and cultured with medium supplemented with 10% FBS and 1% penicillin-streptomycin solution for 24 h. Then, the above cells were incubated with the dipping solution of CaF_2_, Mg-MOF and CMPA (200 µg mL^− 1^), as well as receptor activator of nuclear factor kappa-B ligand (RANKL, 50 ng mL^− 1^) that could facilitate the differentiation of RAW264.7 cells into osteoclast-like cells. After cultivation for 5 days, the original medium in the well plate was removed, and the TRAP fixative was added and fixed at 4℃ for 1 min. After washing twice with PBS, the prepared TRAP staining solution was added to the cells and cocultured at 37℃ for 1 h in the dark. Finally, TRAP-positive osteoclasts in each group were observed using a microscope (LEICA DMi1, China). Alternatively, we extracted RNA from RAW264.7 cells in each group by TRIzol method, and then detected the fluorescence signal by fluorescence quantitative polymerase chain reaction instrument (CFX Connect, USA). The expression levels of osteoclast-related genes in different groups were quantified by quantitative real-time polymerase chain reaction (qRT-PCR) assay, including matrix metalloproteinase 9 (MMP9) and nuclear factor of activated T cells 1 (Nfatc1). All primer sequences were described in Table S1.

### Intracellular reactive oxygen species (ROS) detection

Intracellular ROS were detected by DCFH-DA which could be oxidized by ROS to fluorescent 2,7-dichlorofluorescein. In detail, RAW264.7 cells were seeded in 24-well plates at a density of 2 × 10^4^ cells per well, followed by incubation for 24 h. Then, DMEM containing 10 µg mL^− 1^ lipopolysaccharide (LPS) and different samples (200 µg mL^− 1^) were replaced with the culture medium and co-cultured for 12 h. Afterwards, 10 µM DCFH-DA was added and incubated in darkness for 20 min, and the production of intracellular ROS was monitored by an inverted fluorescence microscope. Furthermore, the relative fluorescence intensity of each group was measured utilizing a multifunctional microplate reader, where the excitation wavelength and emission wavelength were 488 nm and 525 nm respectively.

### Anti-inflammatory study

To assess the anti-inflammatory abilities of the diverse samples, the levels of pro-inflammatory factors including interleukin-6 (IL-6) and tumor necrosis factor-α (TNF-α), along with anti-inflammatory factors containing interleukin-10 (IL-10) and transforming growth factor-β (TGF-β) were measured *via* ELISA. Initially, RAW264.7 cells were plated at a density of 1 × 10^6^ cells per well in a six-well plate, following DMEM with 10% FBS incubated for 24 h, and then preconditioned with diverse samples (200 µg mL^− 1^) for another 24 h. Subsequently, cells were stimulated with 10 µg mL^− 1^ LPS for 12 h, and the cell culture supernatant was harvested through centrifugation (2,000 rpm, 10 min) to estimate the expression of inflammatory factors according to the protocol of the ELISA kit.

### Macrophage polarization assessment

To visualize the effect of the sample on macrophage polarization, an immunofluorescence staining experiment was performed. In a six-well plate, 1 × 10^6^ RAW264.7 cells were seeded per well and incubated with DMEM containing 10% FBS for 24 h. Then, RAW264.7 cells treated with CaF_2_, Mg-MOF and CMPA (200 µg mL^− 1^) were activated with LPS (10 µg mL^− 1^) for 12 h. After washing with PBS for three times, cells were fixed with 4% paraformaldehyde for 10 min and blocked with 3% bovine serum albumin (Servicebio, China) for 30 min. Subsequently, cells were separately stained with fluorescent-conjugated CD86 antibody (Servicebio, China) as M1 marker and CD206 antibody (Servicebio, China) as M2 marker for 1 h at room temperature. Meanwhile, nuclei were counterstained with 4’,6-diamidino-2-phenylindole (DAPI). Eventually, the fluorescence signal was captured on an inverted fluorescence microscope. Alternatively, RAW264.7 cells were subjected to pretreatment according to the above method, and these cells were collected to analyze the expression of CD86 and CD206 by western blot (WB).

### Hemocompatibility analysis

Typically, the fresh whole blood was harvested, centrifuged at 1,500 rpm for 15 min, and rinsed with normal saline three times to obtain red blood cells (RBCs). Then, 100 µL diluted RBCs was mixed with 1.1 mL CaF_2_, Mg-MOF and CMPA solutions (the final working concentrations of the samples were 200 µg mL^− 1^), incubated in a water bath (37℃) for 3 h and centrifuged at 1,500 rpm for 15 min to obtain the supernatant. In a parallel study, 100 µL RBCs was added to 1.1 mL saline solution and 1.1 mL deionized water as the negative control (−) and the positive control (+), respectively. Finally, the supernatant (100 µL) was aspirated into 96-well plates for analyzing the absorbance at a wavelength of 540 nm by a microplate reader. The following equation was utilized to calculate the hemolysis rate:

$$\text { Hemolysis rate (\%) }=\frac{O D_s-O D_n}{O D_p-O D_n} \times 100 \%$$

where OD_s_ was the OD value of the CaF_2_, CM or CMPA group, OD_n_ was the OD value of the negative control group, and OD_p_ was the OD value of the positive control group.

Especially, the RBCs precipitates processed by various methods as mentioned above were collected and gently washed three times with PBS. After fixation with 2.5% glutaraldehyde for 3 h, RBCs were dehydrated with different concentrations of ethanol (30%, 50%, 70%, 80%, 90% and 100%, v/v). Subsequently, the mixture of ethanol and tert-butanol (1: 1, v/v), as well as tert-butanol, were used to replace the above solution in sequence. Ultimately, the morphology of RBCs was imaged using SEM.

### Cell migration assays

HUVECs and MC3T3-E1 cells (5 × 10^5^ cells per well) were seeded into 6-well plates and incubated with DMEM containing 10% FBS for 24 h. Parallel scratches were performed in the well with a pipette, and the floating cells were removed by washing with PBS. Thereafter, the serum-free medium containing CMPA (200 µg mL^− 1^) was used for cultivation, with the medium containing no materials as the control (Ctrl) group. At 0, 12 and 24 h, the migration effect of cells in each group was monitored *via* a microscope, and the scratch width at the corresponding time point was measured through Image-pro Plus J software.

### Assessment of osteogenic activity

MC3T3-E1 cells were seeded with 5 × 10^3^ cells per well in six-well plates and cultured with osteogenic ingredients (10 mM ascorbic acid, 1 M sodium β-glycerophosphate and 1 mM dexamethasone) medium supplemented with the impregnation solution of samples (200 µg mL^− 1^) for 14 days. After coculture, MC3T3-E1 cells were fixed with 4% paraformaldehyde for 10 min and washed three times with PBS, following the manufacturer’s instructions for ARS and ALP staining kits. The images of stained samples were captured by a microscope and quantitatively analyzed using Image-pro Plus J software for analysis of ARS and ALP positive areas. Moreover, MC3T3-E1 cells were cultured with the same method for 14 days, which were collected to estimate the expressions of osteogenetic genes including runt-related transcription factor 2 (RUNX2) and osteocalcin (OCN) through qRT-PCR and WB assays severally, with primers as shown in Table S1.

### In vivo therapy of osteoporosis

The experimental procedures were carried out accordingly with the institutional guidelines for animal care and approved by the animal ethics committee of Nanchang University (Nanchang, China, NCULAE-20221228021). Female KM mice (8-week-old) were purchased from Hunan SJA Laboratory Animal Co., Ltd. (Changsha, China) and stabilized in a room free of specific pathogens for two weeks, allowing access to sufficient food and water. Subsequently, mice underwent bilateral ovariectomy (OVX) under general anesthesia to induce osteoporosis following the previously reported method to estimate the anti-osteoporosis effect of CMPA in vivo [29]. At the same time, KM mice were subjected to sham surgery, which involved removing some adipose tissue with ovaries left untouched, serving as the Sham group. After that, the above animals were randomly assigned to five groups (*n* = 5), including the Sham group, Saline group, CaF_2_ group, Mg-MOF group and CMPA group. Four weeks after surgery, the Sham group and Saline group were intraperitoneally injected with saline (0.9% sodium chloride) as controls, and mice in the other groups were administered with CaF_2_, Mg-MOF or CMPA (200 µg mL^− 1^) through intraperitoneal injection every two days for a total of four weeks. Over eight weeks, the body weight of animals was recorded once a week. At the endpoint, all mice were euthanized by inhalation of isoflurane after diverse treatments, and then the muscles, ligaments and other soft tissues surrounding the bone tissue were stripped to harvest the distal femur. In order to evaluate the therapeutic effect of various groups, the collected bone tissues were fixed in 4% paraformaldehyde for micro-computed tomography (micro-CT) analysis to detect trabecular microarchitecture. Afterward, the bone parameters containing bone mineral density (BMD), bone volume per tissue volume (BV/TV), trabecular number (Tb.N), trabecular separation (Tb.Sp) and trabecular thickness (Tb.Th) were measured by three-dimensional reconstruction using NRecon software.

### In vivo biosafety of CMPA

For assessing the biosecurity of CMPA in vivo, the internal organs (heart, liver, spleen, lung and kidney) dissected from the postoperative mice were collected to conduct hematoxylin and eosin (H&E) staining. Simultaneously, the whole blood and blood serum of mice were obtained severally for the blood routine test and the analysis of blood biochemical indexes, including ALP, alanine transaminase (ALT) and aspartate transaminase (AST).

### Biodistribution study

The typical fluorescent dye RhB was configured into the RhB aqueous solution with a concentration of 2 mg mL^−1^, and 50 mg CMPA was added and stirred overnight. Subsequently, RhB-labeled CMPA was collected by centrifugation and washed several times. Following injection with RhB-labeled CMPA (20 mg kg^−1^), the animals were sacrificed at specific time points (1, 3, 6, 12 and 24 h), while isolated organs (heart, liver, spleen, lung and kidney) in conjunction with lower limb bones were harvested. For monitoring the biological distribution of CMPA in the body, the IVIS imaging system was used to obtain the corresponding fluorescence pictures (Ex: 555 nm and Em: 580 nm).

### Statistical analysis

All data were statistically analyzed with the GraphPad Prism 8.0.1 and expressed as the means ± standard deviation (s.d.) for each group. All measurements were performed at least in three replicates. Statistical analysis was conducted using one-way analysis of variance (ANOVA) followed by *t* test. When the *p*-value was less than 0.05, the differences were considered to be statistically significant and indicated with an asterisk (*p < 0.05, **p < 0.01, ***p < 0.001).

## Result and discussion

### Preparation and characterization of CMPA

**Fig. 1:**
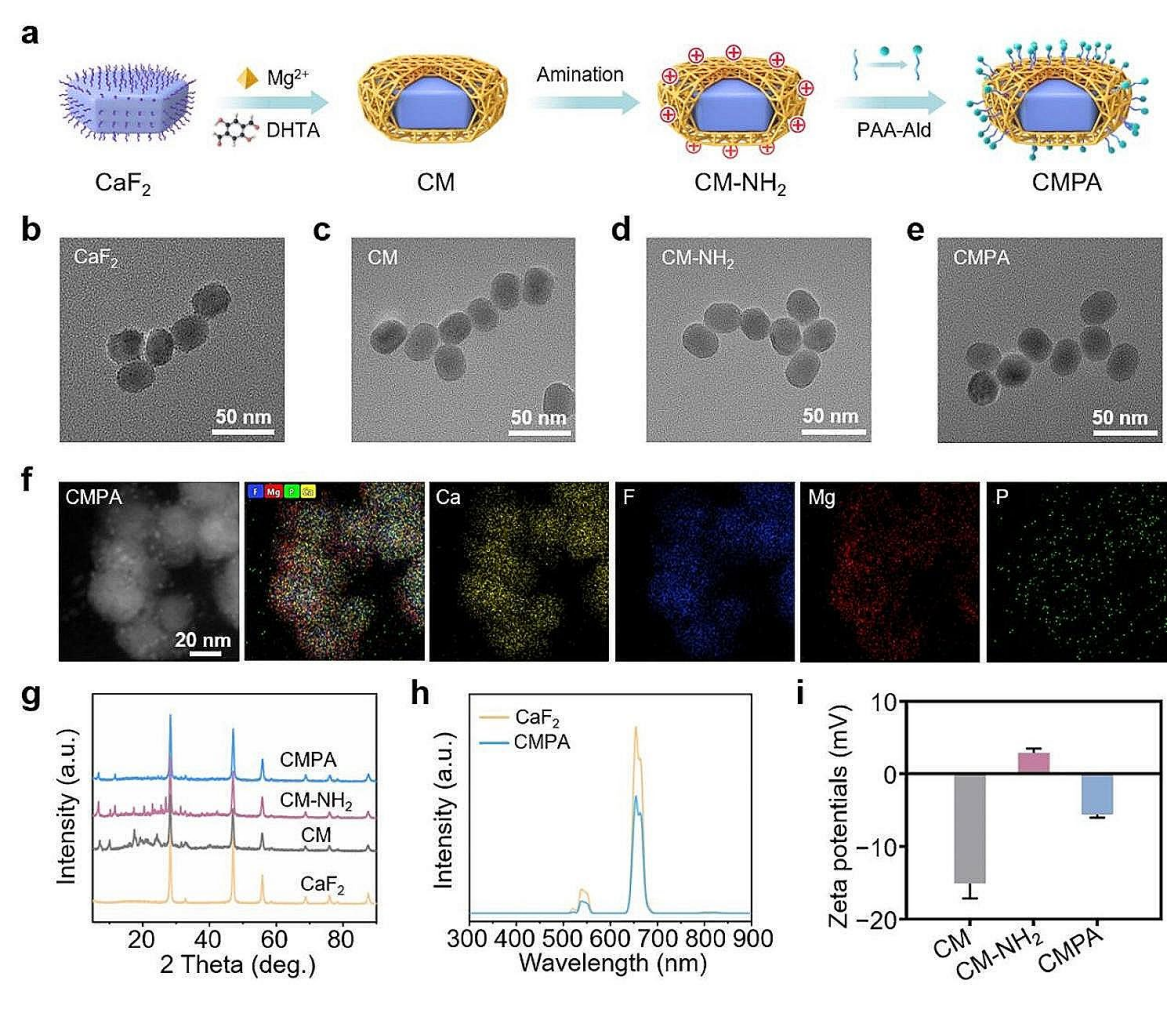
Formation and characterization of CMPA. (**a**) Schematic of the synthesis process of CMPA. (**b-e**) TEM images of CaF_2_, CM, CM-NH_2_ and CMPA. (**f**) The element mapping images of CMPA. (**g**) XRD patterns of CaF_2_, CM, CM-NH_2_ and CMPA. (**h**) The luminescence spectra of CaF_2_ and CMPA. (**i**) Zeta potentials diagram of CM, CM-NH_2_ and CMPA. Data are means ± s.d. (*n* ≥ 3)

As displayed in Fig. 1a, we synthesized and characterized a mineral ion supplementation platform CMPA with bone-targeting function utilizing calcium-based upconversion nanoparticles and magnesium organic framework (Mg-MOF). The core-structured calcium fluoride upconversion nanoparticles (CaF_2_) were synthesized by a simple hydrothermal method previously reported with some modification [22]. The monodisperse CaF_2_ exhibited homogeneously dispersed ellipsoidal forms with a particle size of approximately 28 nm (Fig. 1b and S1a). Subsequently, a layer of Mg-MOF was further deposited on its surface to obtain CaF_2_/Mg-MOF (CM) nanoparticles, and the relevant transmission electron microscopy (TEM) image showed that CM had an ellipsoidal structure with an average size of around 30 nm (Fig. 1c and S1b). To endow CM with a bone-targeting effect, CM was functionalized with alendronate sodium (Ald) possessing a high bone tissue affinity [30]. Specifically, we first performed surface amination of CM (CM-NH_2_) and found no obvious changes in the morphology and size of the aminated nanoparticles (Fig. 1d and S1c). The classic ninhydrin reaction revealed that CM-NH_2_ reacted with the ninhydrin chromogenic agent under heating conditions to generate purple products, elaborating the success of amino modification (Fig. S2) [31]. Meanwhile, Ald was anchored on the surface of polyacrylic acid (PAA) to construct a bone-targeting ligand (PAA-Ald), which was adsorbed to the surface of CM-NH_2_ through electrostatic adsorption, thereby preparing CM-NH_2_-PAA-Ald (CMPA) nanoparticles with bone-targeting efficacy (Fig. 1e and S1d) [28]. Furthermore, element mapping images elucidated the uniform distribution of the major elements of CMPA, including the calcium (Ca) and fluorine (F) elements of CaF_2_, the magnesium (Mg) element of Mg-MOF as well as the specific phosphorus (P) element in bone-targeting groups (Fig. 1f and S3). X-ray diffraction (XRD) was also executed to investigate the crystalline structure of CMPA. As illustrated in Fig. 1g, the characteristic peaks of CaF_2_ and Mg-MOF were observed in CMPA. Thereafter, the luminescence of CMPA under near-infrared laser irradiation at 980 nm was mainly concentrated in green light (about 540 nm) and red light (about 654 nm) derived from the fluorescence spectrums, meaning a certain potential for in vivo imaging (Fig. 1h). Compared with CaF_2_, the fluorescence intensity of CMPA decreased at approximately 540 nm and 654 nm, indicating that the modification of Mg-MOF and PAA-Ald reduced its luminous brightness to some extent. According to X-ray photoelectron spectroscopy (XPS) spectra, it was known that Mg-MOF and bone-targeting ligand PAA-Ald were successfully modified on CaF_2_, indicated by the presence of Mg 1s peak and P 2p peak (Fig. S4). The formation of chemical bonds was confirmed *via* Fourier transform infrared spectroscopy (FTIR). FTIR spectra illuminated that CMPA contained typical absorption peaks of aromatic rings near 1450 cm^− 1^ and 820 cm^− 1^, along with the peak at 1100 cm^− 1^ corresponding to phosphorus-oxygen bonds (Fig. S5). The zeta potentials of CM, CM-NH_2_ and CMPA were also measured. Remarkably, after the electropositive CM-NH_2_ combined with PAA-Ald, the resultant zeta potential of CMPA presented a negative surface charge of approximately − 5.8 mV, which similarly proved the successful modification of bone-targeting ligand Ald (Fig. 1i). In addition, the relative composition of the products at each stage was determined by thermogravimetric analysis (TGA) in Fig. S6, thus manifesting that the materials were fabricated successfully.

### Bone-targeting assessment of CMPA

**Fig. 2:**
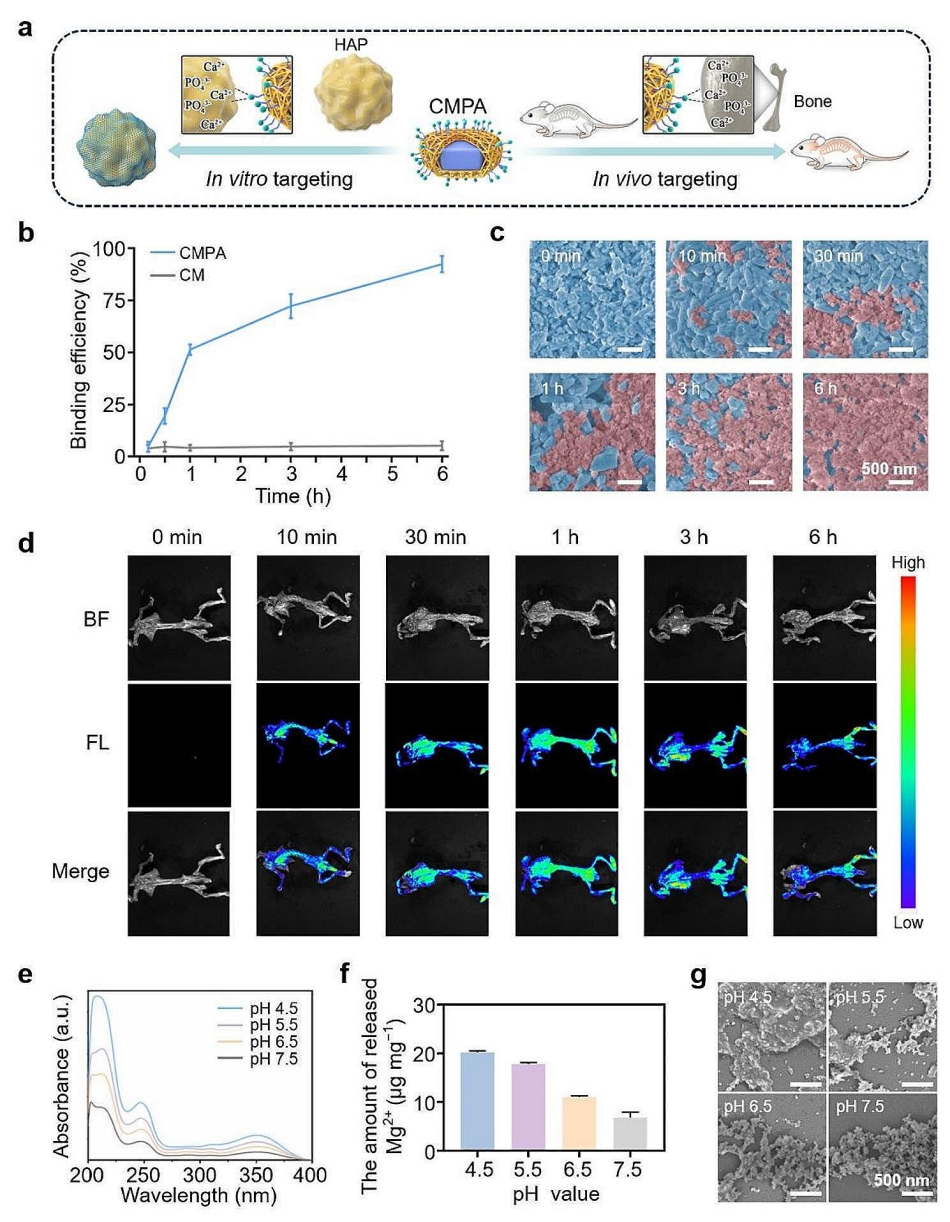
The targeting performance and acid responsiveness of CMPA. (**a**) The schematic diagram of CMPA targeting HAP in vitro and targeting bone tissue in vivo. (**b**) The in vitro binding efficiency of CM and CMPA to HAP at different time intervals. (**c**) Representative SEM images of the interaction between the surface of CMPA and HAP at various time points. (**d**) In vivo fluorescence images at diverse times post-injection of CMPA in mice. (**e**) The ultraviolet-visible (UV-vis) absorption spectra of CMPA at different pH values determined by the released 2,5-dihydroxyterephthalic acid (DHTA). (**f**) The total amount of Mg^2+^ released by CMPA in phosphate buffer solution (PBS) with various pH values. (**g**) The SEM images of CMPA after diverse treatments. Data are means ± s.d. (*n* ≥ 3)

Nanotherapeutic agents have the function of targeting bone tissue and play a critical role in the precise regulation of the microenvironment of osteoporosis [32]. Therefore, achieving effective bone-targeted delivery of CMPA is one of the pivots here. As mentioned earlier, the modification of Ald could endow CM with certain bone-targeting properties, mainly attributed to the chelation of phosphonate groups in Ald chelated with bone calcium to form a bidentate-like structure [33], thereby demonstrating superior bone tissue affinity (Fig. 2a). Initially, hydroxyapatite (HAP), an ideal material for imitating bone tissue, was selected as the target for selective bone delivery of therapeutic drugs to explore the bone-targeting characteristics of CMPA in vitro [34]. As signified in Fig. 2b, the binding efficiency of CMPA to HAP substantially elevated in a time-dependent manner, with a targeting ratio of 92.4 ± 3.9% at 6 h, which was much higher than that of CM. Meanwhile, precipitates were collected at different times and characterized through a scanning electron microscope (SEM) to further evaluate the bone affinity in vitro. The SEM images in Fig. 2c exhibited that CMPA nanoparticles were gradually bound to HAP over time, verifying the good osteo-affinity of CMPA (Fig. S7). However, after 6 h of incubation, there was almost no accumulation of CM on the HAP surface (Fig. S8). Afterwards, the skeletal targeting performance of CMPA in vivo was validated. In short, fluorescein 5-isothiocyanate (FITC)-labeled CMPA was injected into mice, and the in vivo imaging system (IVIS) was used to track the internal distribution of CMPA for various durations. Fluorescence images showed that with the extension of time, the fluorescence signal on the mouse skeleton emerged in a trend of gradually increasing and then decreasing, which confirmed that CMPA could target bone tissue (Fig. 2d and S9). The above results clarified that CMPA had a favorable skeleton targeting ability, which would assist in the targeted treatment of osteoporosis in vivo.

### The pH responsiveness and osteoclast inhibition activity of CMPA

Osteoporosis is a disease featuring imbalanced bone remodeling. During the absorption stage, a large number of mature osteoclasts adhere to the bone surface and secrete hydrogen ions (H^+^) to acidify the local bone microenvironment, which in turn leads to the dissolution of the mineral phase of the bone [2]. Therefore, the release of key therapeutic ions in the acidic microenvironment (pH ∼ 4) of osteoporosis can be implemented by utilizing the distinguishing feature of pH signal responsiveness to exert the corresponding curative effect. To examine the pH-responsive function of CMPA, it was dispersed in PBS with different pH values to measure the release of ligand (DHTA) and magnesium ions (Mg^2+^) contained in Mg-MOF. As revealed by Fig. 2e, the amount of DHTA gradually increased depending on the decrease of pH, indicating a faster degradation rate of CMPA at low pH. In addition, the content of Mg^2+^ during the degradation of CMPA in response to acidity was detected by an inductively coupled plasma (ICP) emission spectrometer. In an acidic milieu (pH 4.5), the amount of released Mg^2+^ was higher than that of other groups (Fig. 2f). Concomitantly, the representative SEM images indicated that, unlike the ellipsoidal morphology of CMPA under pH 7.5, it underwent clear collapse after immersing in acidic PBS (pH 4.5), accompanied by the release of calcium ions (Ca^2+^), confirming the pH response capacity of CMPA (Fig. 2g and S10).

Subsequently, we further studied the effect of CMPA on osteoclast viability. Mouse macrophage RAW264.7, as a crucial precursor of osteoclasts, can be induced to osteoclast differentiation in the presence of cytokines such as receptor activator of nuclear factor kappa-B (NF-κB) ligand (RANKL) [35, 36]. For the osteoclast differentiation research, RAW264.7 cells were cultured with RANKL (50 ng mL^− 1^) supplemented with CMPA for 5 days, followed by tartrate resistant acid phosphatase (TRAP) staining to evaluate the formation of osteoclasts in each group. The outcomes validated that both the number and size of TRAP-positive multinucleated osteoclasts in the CaF_2_, Mg-MOF and CMPA groups were reduced compared with the RANKL group (Fig. S11a). Furthermore, a quantitative real-time polymerase chain reaction (qRT-PCR) assay was chosen to determine the expression of osteoclast related genes like bone resorption enzyme (matrix metalloproteinase 9, MMP9) and osteoclast transcription factor (nuclear factor of activated T cells 1, Nfatc1) [37]. According to Fig. S11b and S11c, a significant decrease in the levels of osteoclast-specific functional genes (MMP9 and Nfatc1) was seen in the CMPA-treated group, which further manifested that CMPA could efficaciously suppress the activity of osteoclasts. Overall, CMPA was capable of responding to the highly enriched acidic microenvironment constructed by osteoclasts, diminishing osteoclast formation and interfering with bone resorption, which was conducive to the regulation of acidic osteoporotic bone microenvironment and the effective treatment of osteoporosis.

### Anti-inflammation effect of CMPA

**Fig. 3:**
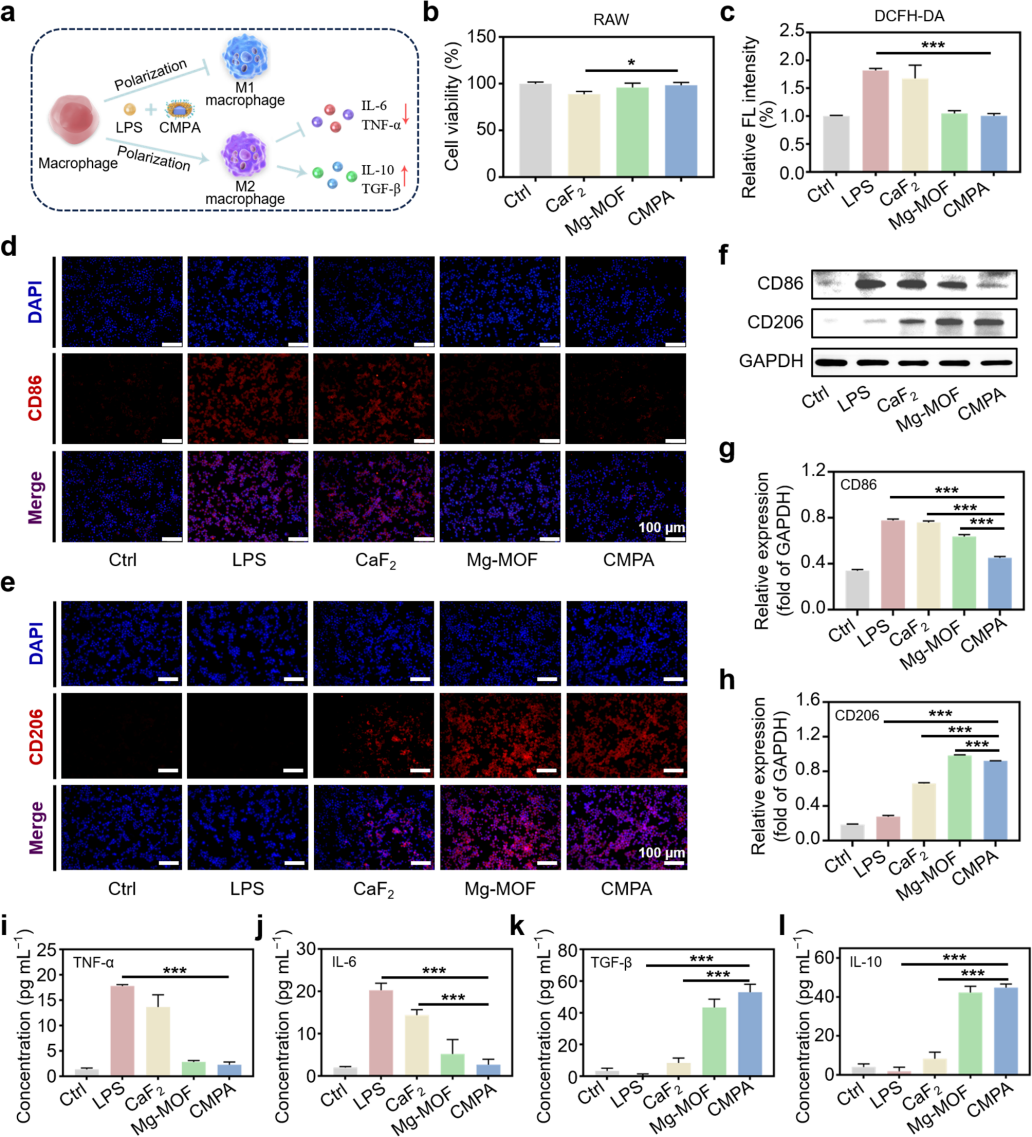
The anti-inflammatory ability of CMPA. (**a**) The scheme of inflammation regulation of CMPA. (**b**) Cell viability of RAW264.7 cells with diverse treatments. (**c**) The relative fluorescence intensity of RAW264.7 cells stained by 2’,7’-dichlorofluorescein diacetate (DCFH-DA) in different groups. (**d**) Immunofluorescence images of the expression of CD86 in RAW264.7 cells with various treatments. (**e**) Immunofluorescence images of the expression of CD206 in RAW264.7 cells in diverse groups. (**f**) WB detection results of inflammation-related protein expression. (**g, h**) The protein expression level of CD86 and CD206. (**i-l**) ELISA assays of TNF-α, IL-6, TGF-β and IL-10 in LPS-induced RAW264.7 cells after different treatments. Data are means ± s.d. (*n* ≥ 3). *p < 0.05, **p < 0.01, ***p < 0.001

Chronic inflammation is the primary contributor to osteoporosis, so manipulating the inflammatory microenvironment induced by macrophages is equally important for the therapy of osteoporosis [27]. Typically, macrophages exhibit two major phenotypes in accordance with the specific environment, with respect to the classically activated macrophages’ (M1) polarized pro-inflammatory phenotype and alternatively activated macrophages’ (M2) polarized anti-inflammatory phenotype (Fig. 3a) [9]. The transformation of the initial inflammatory M1-type macrophages to anti-inflammatory M2-type macrophages is beneficial for the reconstruction of normal bone homeostasis. Herein, RAW264.7 cells acted as an inflammatory cell model to assess the immunomodulatory efficacy of CMPA. We preliminarily bore out the effect of the samples on the activity of RAW264.7 cells, and cell counting kit-8 (CCK-8) test results showed that CMPA had almost no toxicity to cells (Fig. 3b). Moreover, CMPA could resist endogenous oxidative stress induced by lipopolysaccharide (LPS) to a certain extent (Fig. 3c and S12), which was expected to alleviate the inflammatory response mediated by reactive oxygen species (ROS) [38]. Afterward, a detailed exploration was conducted on the immune regulatory ability of CMPA to guide macrophage polarization from the M1 phenotype to the M2 phenotype. The polarization status of macrophages under LPS stimulation was evaluated by immunofluorescence staining and western blot (WB) testing, with two typical macrophage markers including CD86 (M1 marker) and CD206 (M2 marker) serving as reference indicators [39]. As illustrated in Fig. 3d and e, CD86 was found to be strongly expressed in the proinflammatory macrophage stage (M1) in the LPS group, indicating that the LPS-provoked inflammatory microenvironment was successfully constructed. On the contrary, less CD86 and more CD206 fluorescence signals were observed in RAW264.7 cells treated with CMPA, which were equivalent to the inflammatory inhibition of Mg-MOF, possibly ascribed to the good anti-inflammatory performance of Mg^2+^ [15]. Meanwhile, the WB detection results were consistent with immunofluorescence staining, further proving that CMPA had the characteristics of regulating macrophage polarization (Fig. 3f-h). Additionally, the secretion of representative inflammatory cytokines was measured using enzyme-linked immunosorbent assay (ELISA), which contained pro-inflammatory factors tumor necrosis factor-α (TNF-α) and interleukin-6 (IL-6), along with anti-inflammatory factors transforming growth factor-β (TGF-β) and interleukin-10 (IL-10). We found that the level of TNF-α and IL-6 was noticeably downregulated in the CMPA group (Fig. 3i and j), whereas the expression of TGF-β and IL-10 was decidedly enhanced (Fig. 3k and l), thus achieving the purpose of mitigating inflammation. The above results all testified that CMPA could suppress the production of M1-type macrophages and the level of related pro-inflammatory cytokines (TNF-α and IL-6), thus disrupting the inflammatory microenvironment beneficial to osteoclast growth [40]. Concurrently, CMPA was capable of augmenting the secretion of TGF-β and IL-10 by anti-inflammatory M2-type macrophages, which was implicated in the promotion of bone tissue repair and regeneration [27].

### Anti-inflammation mechanism of CMPA

**Fig. 4:**
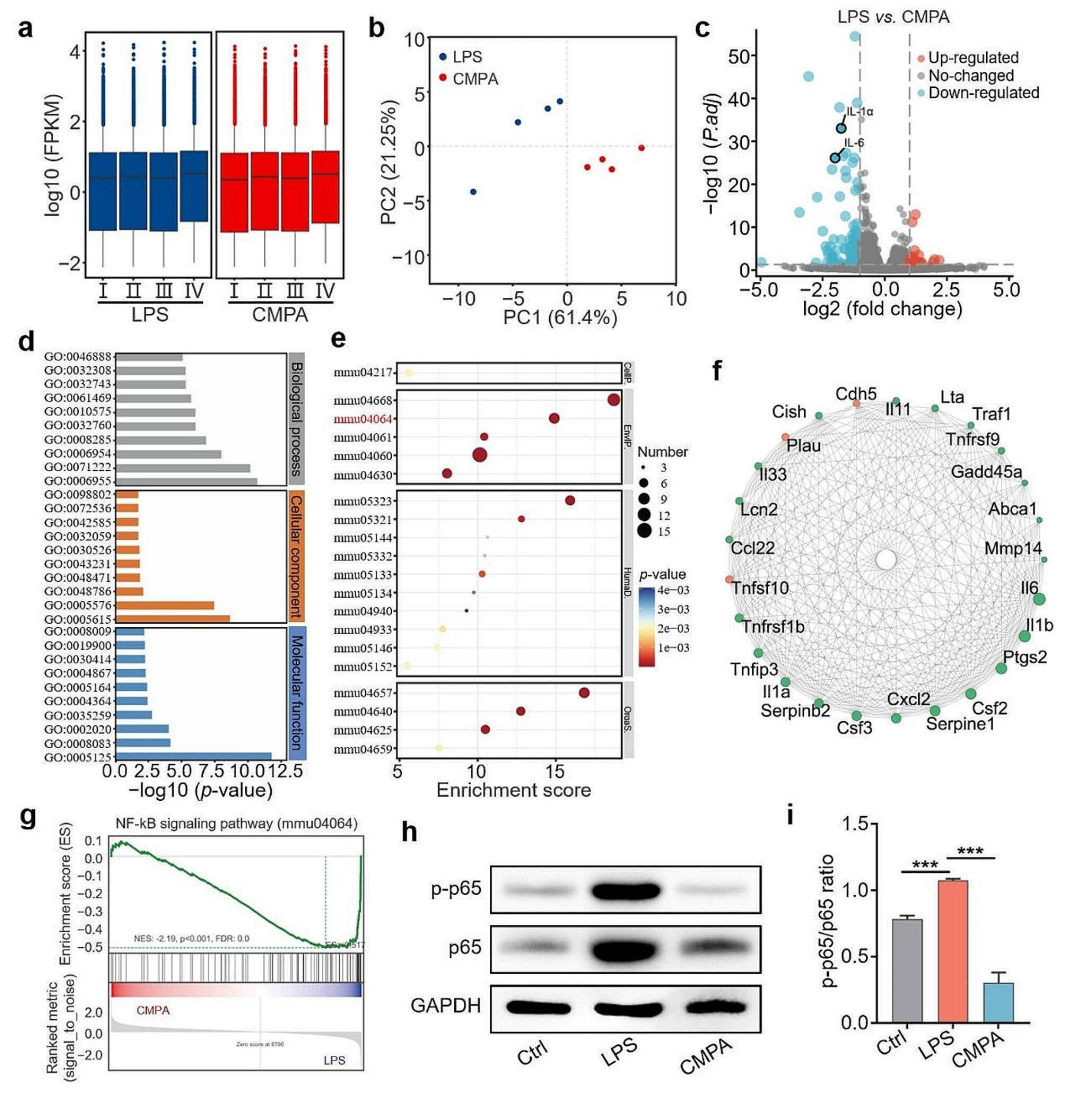
The anti-inflammation mechanism of CMPA. (**a**) The boxplot data of different groups. (**b**) PCA results of the LPS and CMPA groups. (**c**) Volcano plots of DEGs in RAW264.7 cells with various treatments. (**d**) GO analysis of the down-regulated DEGs in the top 30. (**e**) KEGG pathways enrichment analysis of the down-regulated DEGs in the top 20 between the LPS and CMPA groups. (**f**) PPI network analysis. Green represented downregulated genes, and orange represented upregulated genes. (**g**) GSEA of the NF-κB signaling pathway. (**h**) WB detection results of p-p65 and p65. (**i**) The p-p65/p65 ratio of diverse groups. Data are means ± s.d. (*n* ≥ 3). *p < 0.05, **p < 0.01, ***p < 0.001

In order to further elucidate the molecular pathways dominating the inflammatory regulation of CMPA, we performed a transcriptome sequencing (RNA-seq) of RAW264.7 cells subjected to diverse treatments. The boxplot displayed the standardization of RNA-seq data, indicating a good homogenization effect of each sample (Fig. 4a). The results of principal component analysis (PCA) revealed a high relevance among samples within the same group, whereas favorable discrimination existed between the LPS and CMPA groups (Fig. 4b). The distribution of differentially expressed genes (DEGs) in various groups was compared by volcano plot, in which gray represented the genes with an insignificant difference, while cerulean and orange represented the genes with a significant difference (Fig. 4c). Based on the statistics of DEGs, there were totally 106 DEGs with statistical significance identified from the gene expression profile of RAW264.7 cells, of which 28 DEGs were upregulated (orange), and 78 DEGs were downregulated (cerulean). Notably, compared with the LPS group, the genes participated in the pro-inflammatory role were considerably downregulated in the CMPA group, such as IL-6, interleukin-1α (IL-1α) and interleukin-1β (IL-1β) [41]. As shown in Fig. 4d, gene ontology (GO) analysis illustrated that the downregulated DEGs in the top 30 were gathered in biological processes, among which representative GO terms contained “inflammatory response” and “immune response” (Table S2). Furthermore, the Kyoto Encyclopedia of Genes and Genomes (KEGG) pathway analysis was conducted on DEGs. It was discovered that after CMPA treatment, the low expression genes were predominantly enriched in “NF-κB signaling pathway”, “tumor necrosis factor (TNF) signaling pathway” and “interleukin-17 (IL-17) signaling pathway”, which were closely associated with the occurrence of inflammatory reactions (Fig. 4e and Table S3) [42, 43]. Correspondingly, the protein-protein interactions (PPI) network diagram also listed the core interacting proteins that were primarily involved in the manipulation of pro-inflammatory genes (such as IL-1α, IL-1β and IL-6), which were the vital factors leading to the activation of NF-κB (Fig. 4f) [44]. Gene set enrichment analysis (GSEA) based on RNA-seq expression matrix emphasized that NF-κB signaling pathway was markedly inhibited in CMPA-treated RAW264.7 cells (NES: −2.19, *p* < 0.001), suggesting that CMPA had the potential to abrogate the LPS-triggered inflammation (Fig. 4g). As well documented, p65 belongs to a subunit of the NF-κB family that can activate NF-κB pathway to induce inflammation [45]. Phospho-p65 (p-p65), a phosphorylated form of p65, can affect the transcriptional activity of p65 [46]. Next, WB assay was utilized to determine the levels of p65 and p-p65 to further verify the inhibitory capability of CMPA on the NF-κB pathway. As depicted in Fig. 4h and i and S13, the expression of p65 and p-p65 proteins was dramatically strengthened in LPS-activated RAW264.7 cells, while CMPA attenuated the phosphorylation of p65 protein, thereby suppressing the activation of NF-κB [47]. Collectively, these results implied that CMPA might exhibit potent anti-inflammatory efficacy by inhibiting the signaling pathway of NF-κB, thus normalizing the pathological microenvironment and enhancing osteogenesis.

### Evaluations of cytocompatibility and osteogenesis of CMPA in vitro

**Fig. 5:**
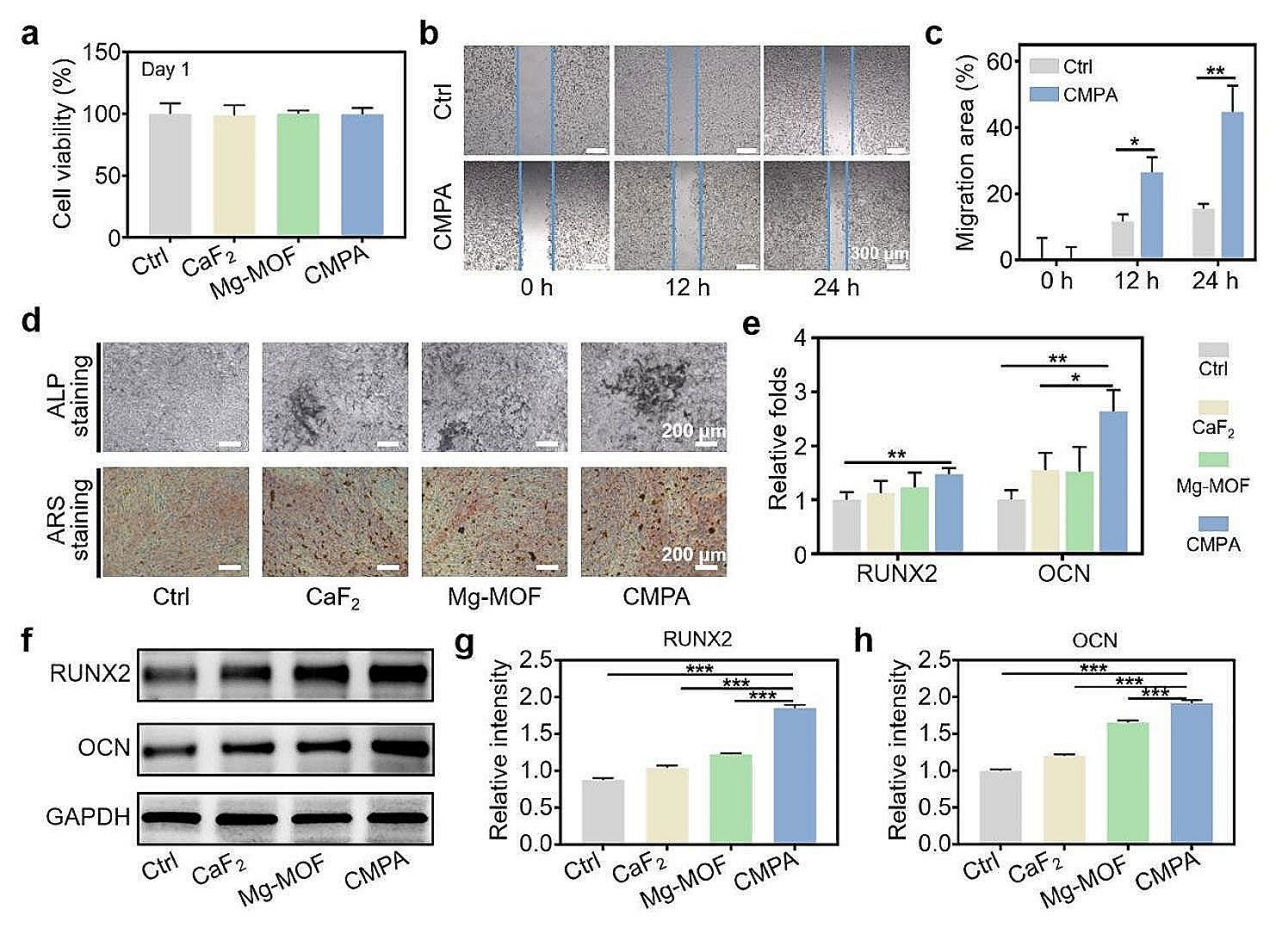
Evaluation of osteogenic properties of CMPA. (**a**) The cytotoxicity of CaF_2_, Mg-MOF and CMPA on MC3T3-E1 cells for 1 day. (**b, c**) Microscopic images and quantitative analysis of MC3T3-E1 cells scratch test in diverse groups. (**d**) ALP and ARS staining of MC3T3-E1 cells with various treatments. (**e**) Relative mRNA expression of RUNX2 and OCN detected by qRT-PCR. (**f-h**) The level of RUNX2 and OCN detected by WB analysis and their relative intensity quantification. Data are means ± s.d. (*n* ≥ 3). *p < 0.05, **p < 0.01, ***p < 0.001

The biosecurity and osteogenesis of CMPA are indispensable for the therapeutic outcome of osteoporosis [48]. Firstly, mouse pre-osteoblast cells (MC3T3-E1), human bone marrow mesenchymal stem cells (hMSCs) in conjunction with human umbilical vein endothelial cells (HUVECs) were chosen to validate the cytocompatibility of CaF_2_, Mg-MOF and CMPA through CCK-8, flow cytometric analysis and live/dead cell staining experiments. Our finding also demonstrated that CMPA bespoke negligible cytotoxicity in MC3T3-E1 cells, even after 3 days of co-culture at a dose as high as 200 µg mL^− 1^ (Figs. 5a, S14 and S15). It was noteworthy that the activity of hMSCs and HUVECs cells in the CMPA group was higher than that of the CaF_2_ group, implying that the modification of Mg-MOF improved cell compatibility to some degree (Figs. S16 and S17). Simultaneously, the hemolysis rates of various samples were less than 5%, and there was no significant impact on the morphology of red blood cells (RBCs), which attested to good hemocompatibility (Fig. S18). To preliminarily assess the osteogenic efficacy, we investigated the migration of MC3T3-E1 cells treated with CMPA at different time intervals. As described in Fig. 5b and c, compared with the control (Ctrl) group, the migratory capacity in the CMPA group was prominently enhanced, affirming that CMPA potently accelerated the cell migration of MC3T3-E1 cells. Similar to the MC3T3-E1 cells, the migration rate of HUVECs cells increased with prolonged coincubation time, indicating that CMPA was essential for the potential to facilitate angiogenesis in addition to the superiority of osteogenesis (Fig. S19) [49]. Then, the osteogenic behavior of diverse materials in vitro was further verified by alkaline phosphatase (ALP) and alizarin red S (ARS) staining as well as qRT-PCR assays. ALP is a well-known early marker for reflecting osteogenic differentiation, while ARS is the decisive indicator for judging the formation of mineralized nodules in osteoblasts [50]. According to the ALP staining images, the CMPA group showed the highest ALP activity after 14 days of cultivation compared with other groups, and an analogous trend was found in the mineralized nodules of MC3T3-E1 cells stained with ARS (Fig. 5d and S20). Additionally, the expression levels of osteogenic-related genes in the CMPA group were notably higher than those in the Ctrl group, including runt-related transcription factor 2 (RUNX2) and osteocalcin (OCN), followed by the CaF_2_ and Mg-MOF groups (Fig. 5e). Subsequently, the expression of osteogenesis-related proteins (RUNX2 and OCN) in varying groups was comprehensively profiled *via* WB assay, which was similar to the experimental outcomes of qRT-PCR (Fig. 5f-h). Ultimately, all results collectively proved that CMPA, which could supplement both calcium and magnesium, was more conducive to osteogenic differentiation than calcium (CaF_2_) or magnesium (Mg-MOF) alone.

### In vivo antiosteoporosis effect of CMPA

**Fig. 6:**
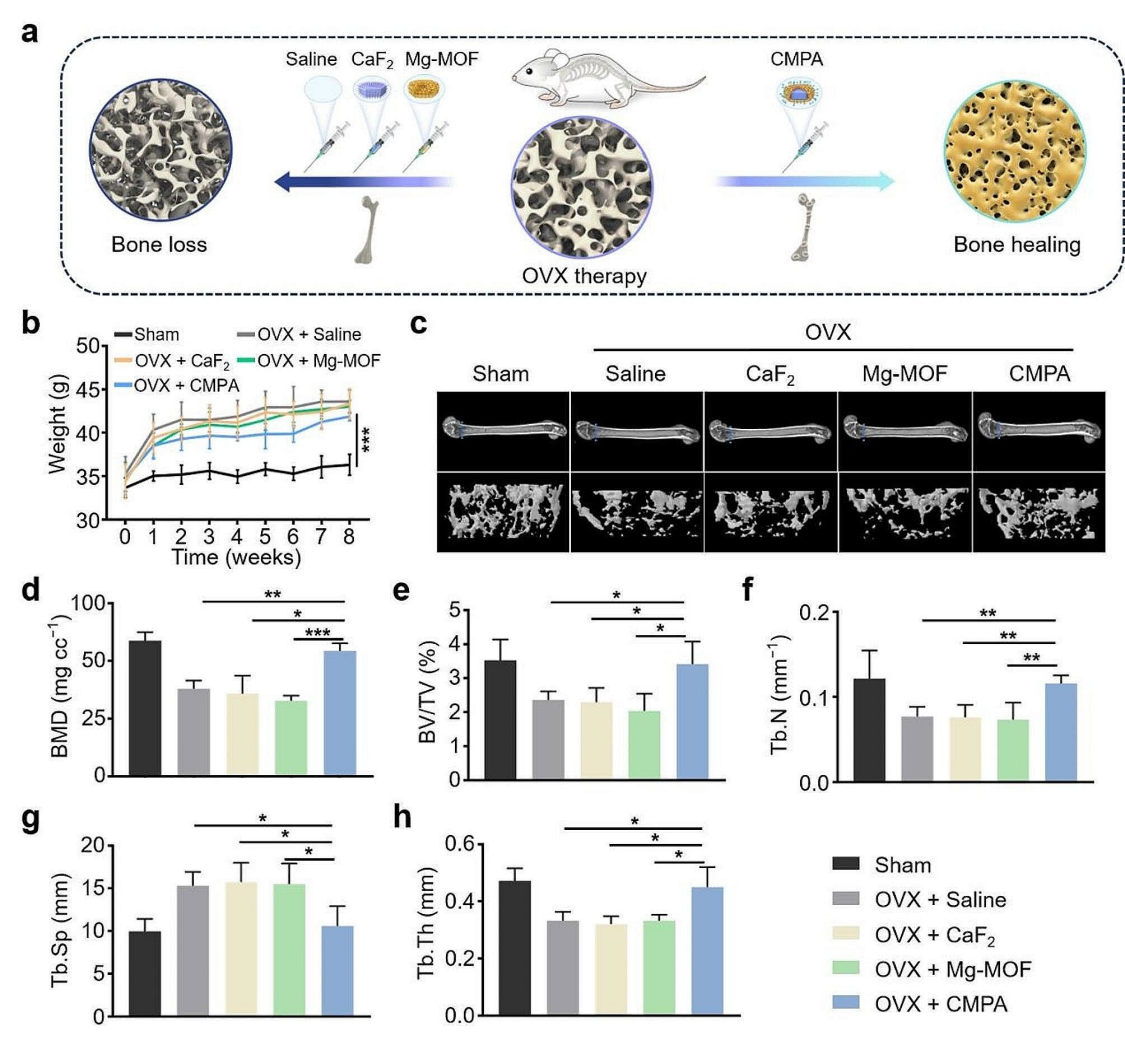
The therapeutic effect of CMPA on osteoporosis in vivo. (**a**) Schematic diagram of diverse nano-preparations for the treatment of osteoporosis mice. (**b**) Body weight changes of mice with various treatments. (**c**) Three-dimensional reconstructed images of the distal femur in diverse groups. (**d-h**) Architectural parameters of BMD, BV/TV, Tb.N, Tb.Sp and Tb.Th of the distal femur in various groups. Data are means ± s.d. (*n* ≥ 3). *p < 0.05, **p < 0.01, ***p < 0.001

In virtue of the potential of CMPA to normalize the inflammatory pathological microenvironment in vitro, as well as its ability to inhibit osteoclast formation and facilitate osteoblast differentiation, a murine model of ovariectomy (OVX)-induced osteoporosis was established to further investigate the effect of CMPA on reversing osteoporotic bone loss [29]. Briefly, the Kunming (KM) mice were either ovariectomized or sham-operated, followed by four weeks of modeling. Afterwards, the OVX mice in each group were intraperitoneally injected with CaF_2_, Mg-MOF or CMPA solution for one month, while the Sham and Saline groups underwent saline (0.9% sodium chloride) treatment as controls (Fig. 6a). During this process, we recorded and estimated the weight changes of mice in diverse groups. Apparently, the weight of the mice in the Sham group remained relatively stable, whereas mice subjected to OVX showed evident weight gain, signifying the successful construction of the osteoporosis model provoked by OVX (Fig. 6b) [28]. At the endpoint, micro-computed tomography (micro-CT) was adopted to observe the recovery of the collected distal femur. As evidenced in Fig. 6c, OVX mice experienced significant bone loss compared with those undergoing sham surgery. After administration of CMPA, the bone loss caused by the OVX procedure was rescued, exhibiting a much denser bone tissue structure, which was superior to CaF_2_ or Mg-MOF. Quantitative analysis of bone parameters manifested that relative to the Sham group, the bone mineral density (BMD), bone volume per tissue volume (BV/TV), trabecular number (Tb.N) and trabecular thickness (Tb.Th) of the spine and femur were decreased in the Saline group, and the trabecular separation (Tb.Sp) was noticeably increased (Fig. 6d-h). Synchronously, there was no obvious bone mass improvement in the CaF_2_ and Mg-MOF groups, possibly due to the lack of targeted function in CaF_2_ or Mg-MOF, making it difficult to accurately and efficiently act on bone tissue to exert efficacy. In contrast, CMPA performed better in restoring BMD, BV/TV, Tb.N and Tb.Th, along with a corresponding reduction in Tb.Sp, almost back to a similar level as the Sham group. The above results indicated that calcium (CaF_2_) or magnesium (Mg-MOF) supplementation conducted in a regular manner to alleviate bone loss in osteoporotic mice was limited in the short term, but targeted delivery of calcium and magnesium (CMPA) to bone tissue exhibited a potent therapeutic effect on osteoporosis.

Alternatively, the in vivo biosafety profile of CMPA was examined in female KM mice for pharmacodynamic studies. After injection of CMPA, the heart, liver, spleen, lung, kidney and lower limb bones were sequentially harvested at 1, 3, 6, 12 and 48 h, and the biodistribution of CMPA in the body was monitored through IVIS. Fluorescence images confirmed that the main accumulation organs outside bone tissue were the liver and kidney, which were the primary organs of metabolism in vivo (Fig. S21). Likewise, the major isolated organs (heart, liver, spleen, lung and kidney) were stained with hematoxylin and eosin (H&E). As displayed in Fig. S22, no discernible injuries or histopathological changes were observed in the H&E staining of tissue sections in the CMPA group. Moreover, the representative hematological parameters of mice treated with CMPA were within the normal range (Table S4), while the typical serum levels of biomarkers relevant to liver function were not considerably abnormal (Fig. S23), concerning ALP, alanine transaminase (ALT) and aspartate transaminase (AST). These preliminary results demonstrated that CMPA had the desired biosecurity, which was an effective nanomedicine for combating osteoporosis.

## Conclusion

In this study, a nano-formulation CMPA with bone-targeting and pH-responsive capabilities is synthesized as a novel therapeutic option for osteoporosis therapy, which can synergistically restore bone homeostasis and precisely deliver calcium and magnesium to ameliorate osteoporosis symptoms. Bone-targeted CMPA can localize within bone tissue and degrade in response to the acidic microenvironment generated by osteoclasts, thus releasing therapeutic ions to downregulate the activity of osteoclasts to interfere in bone resorption. The nano-preparation is capable of promoting the effective polarization of macrophages from the inflammatory M1 phenotype to the anti-inflammatory M2 phenotype. It bears out that CMPA primarily reverses the inflammatory microenvironment by suppressing the secretion of pro-inflammatory factors (TNF-α and IL-6) and potentiating the expression of anti-inflammatory factors (IL-10 and TGF-β), thereby increasing osteoblast activity and inducing bone formation. Further, RNA-seq and WB results elucidate the possible molecular mechanism for inflammatory regulation, which is predominantly to mitigate inflammation by inhibiting the NF-κB signaling pathway. Additionally, CMPA can efficaciously provide calcium and magnesium repletion to augment the proliferation and differentiation of osteoblasts to enhance osteogenic activity, ultimately attenuating bone loss in osteoporotic mice. However, the long-term biocompatibility of the material and the strategy for reducing drug administration frequency remain to be studied. Moreover, the treatment of the osteoporosis model in large animals, including pigs and beagles, should be studied. Meanwhile, the mechanism of promoting bone formation in the combination therapy of calcium and magnesium elements, as well as the better combination plan of the two elements, still need further optimization. In the future, this acid-responsive nanoplatform with bone-targeting and immune microenvironment regulation functions is expected to treat bone disorders with excessive bone loss, such as osteoporosis periodontitis, peri-implant infection, etc.

### Electronic supplementary material

Below is the link to the electronic supplementary material.

Supplementary Material 1

## Data Availability

No datasets were generated or analysed during the current study.
