# Supplementary material for: Inflammatory microenvironment regulation and osteogenesis promotion by bone-targeting calcium and magnesium repletion nanoplatform for osteoporosis therapy
Source: J Nanobiotechnology. 2024 Jun 5;22:314. doi: 10.1186/s12951-024-02581-7 (PMC11151641; doi:10.1186/s12951-024-02581-7)
Supplement: Supplementary file 1 — Supplementary Material 1 [file 12951_2024_2581_MOESM1_ESM.docx]

Supplementary Material

**Inflammatory Microenvironment Regulation and Osteogenesis Promotion by Bone-Targeting Calcium and Magnesium Repletion Nanoplatform for Osteoporosis Therapy**

Zhenzhen Weng^a, b, 1^, Jing Ye^a, c, 1^, Changxiong Cai^a^, Zikang Liu^c^, Yuanyuan Liu^c^, Yingying Xu^c^, Jinghong Yuan^a^, Wei Zhang^b^, Lubing Liu^c^, Junkai Jiang^c^, Xigao Cheng^a*^ and Xiaolei Wang^b, c*^

^a^ Department of Orthopaedics, The 2^nd^ Affiliated Hospital, Jiangxi Medical College, Nanchang University, Nanchang, Jiangxi 330088, P. R. China.

^b^ School of Chemistry and Chemical Engineering, Nanchang University, Nanchang, Jiangxi 330088, P. R. China.

^c^ The National Engineering Research Center for Bioengineering Drugs and the Technologies, Institute of Translational Medicine, Nanchang University, Nanchang, Jiangxi 330088, P. R. China.

^1^ Zhenzhen Weng and Jing Ye contributed equally to this work.

* Corresponding authors.

E-mail addresses: [Ndefy12160@ncu.edu.cn](mailto:Ndefy12160@ncu.edu.cn) (Xigao Cheng), wangxiaolei@ncu.edu.cn (Xiaolei Wang).

**1. Experimental methods**

1.1. Evaluation of Cytocompatibility *in Vitro*

HUVECs, hMSCs, MC3T3-E1 and RAW264.7 cells were selected to investigate the cytocompatibility of samples. Cells were seeded onto 96-well plates at a density of 2 × 10^3^ cells per well and incubated in DMEM containing 10% FBS and 1% penicillin-streptomycin solution for 24 h in a CO_2_ incubator (37℃). Then, the original medium was changed to the impregnation solution of different materials and cultured for 1, 2 and 3 days. After that, the above medium was switched to 100 μL CCK-8 (10%, v/v) for 2 h, and the optical density (OD) value at 450 nm was measured utilizing a multifunctional microplate reader (SpectraMaxM5). The cell viability of cells was calculated by the following formula:

$$Cell viability \left( \% \right) = \frac{\mathrm{OD}}{\mathrm{OD}_{0}} \times100\%$$

where OD represented the absorbance of the sample treatment groups, and OD_0_ corresponded to the absorbance of the control group.

Additionally, the cytocompatibility was further verified *via* live/dead cell staining. In brief, hMSCs and MC3T3-E1 cells were seeded into 24-well plates at the density of 2 × 10^4^ cells per well and cultured for 24 h, followed by the addition of diverse samples. After coincubation for another 24 h, the cells were stained with Calcein-AM and PI, which were then visualized by an inverted fluorescence microscope (IX 83, Olympus, Japan). Concurrently, the viability of hMSCs cells treated with the same treatments was evaluated using flow cytometry analyses. Specifically, the processed hMSCs cells were digested with trypsin, resuspended with PBS, and centrifuged, followed by the removal of the supernatant and collection of cell precipitates. Subsequently, all cells were stained with Annexin-V and PI for 20 min at room temperature in the dark. Finally, hMSCs cells were detected using flow cytometry.

1.2. Transcriptome and Bioinformatics Analyses

To further explore the molecular pathways dominating the inflammation-resolving activity of CMPA, transcriptome sequencing (RNA-seq) analysis was conducted on RAW264.7 cells with diverse treatments. In particular, RAW264.7 cells were seeded into cell culture flasks at an appropriate density and cultured with DMEM containing 10% FBS for 24 h. After that, the original medium was replaced with DMEM supplemented with CMPA impregnation solution (200 μg mL^−1^) for 24 h, and 10 μg mL^−1^ LPS was added to induce the cells for 12 h. Among them, the cells treated without CMPA in the cell culture flasks were set as the LPS group. After culturing, the cells were trypsinized, centrifuged and washed three times with PBS, and RNA was extracted from the cells by adding TRIzol reagent. RNA-seq and bioinformatics analysis were administered by OE Biotechnology Co., Ltd. (Shanghai, China).

**2. Figures and tables**


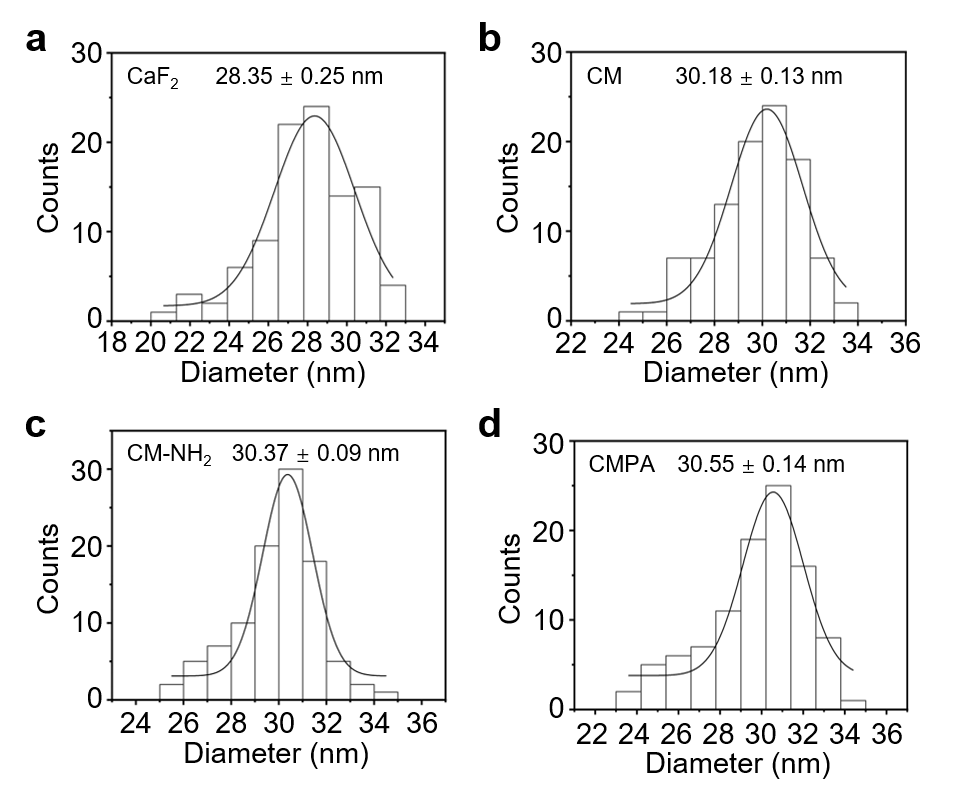


**Figure S1.** (a-d) The particle size distribution of CaF_2_, CM, CM-NH_2_ and CMPA.


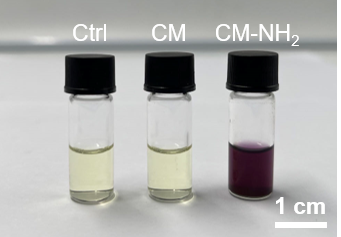


**Figure S2.** The ninhydrin reaction of CM and CM-NH_2_.


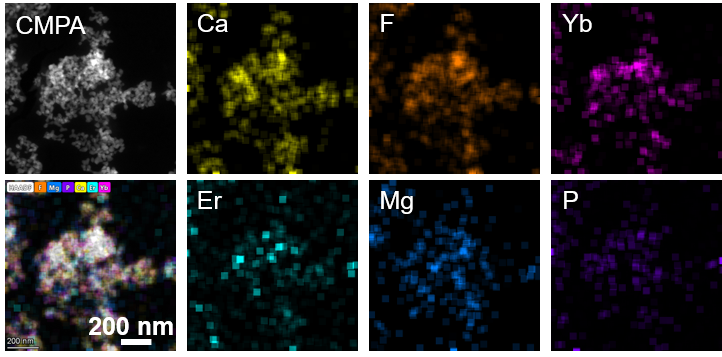


**Figure S3.** Elemental mapping images of CMPA.


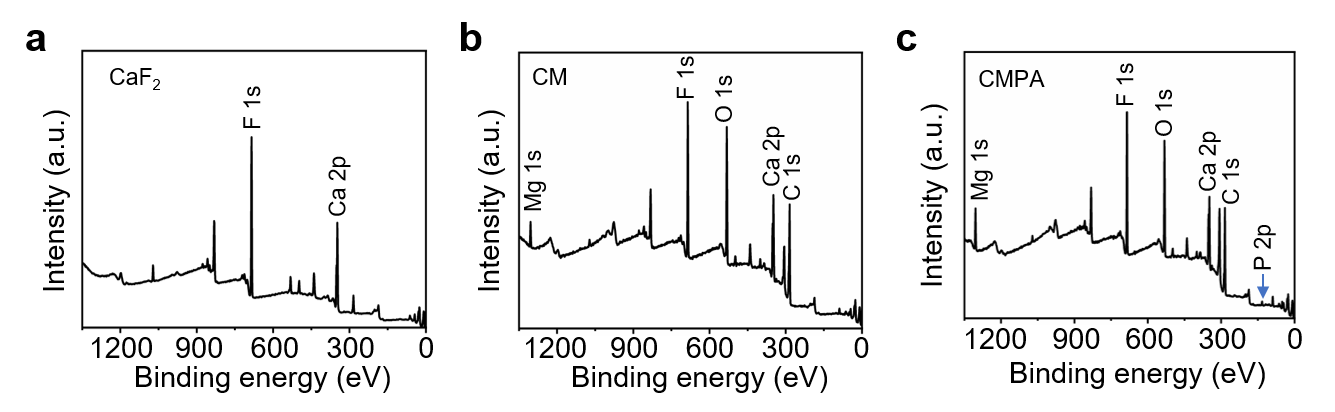


**Figure S4.** (a-c) XPS spectrums of CaF_2_, CM and CMPA.


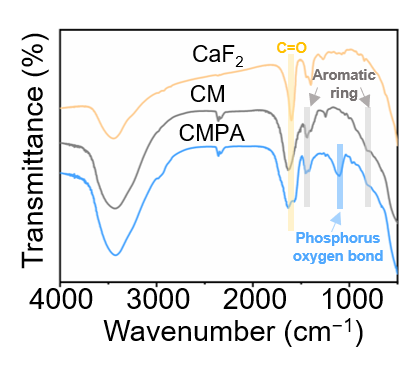


**Figure S5.** FTIR spectra of CaF_2_, CM and CMPA.


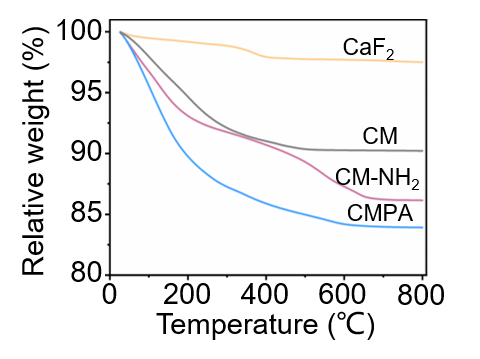


**Figure S6.** TGA data of CaF_2_, CM, CM-NH_2_ and CMPA.


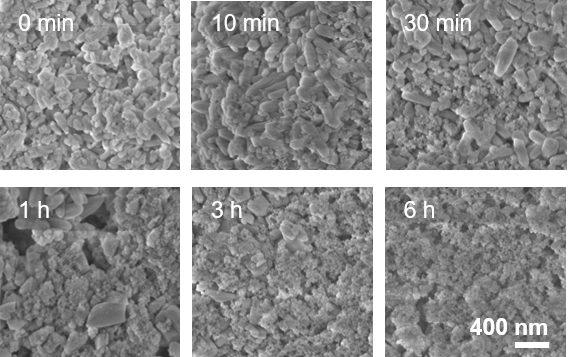


**Figure S7.** Representative SEM images of the interaction between the surface of CMPA and HAP at different time points.


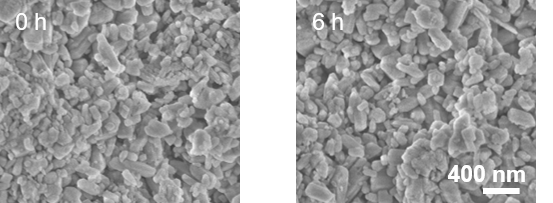


**Figure S8.** Representative SEM images of the interaction between the surface of CM and HAP at diverse time intervals.


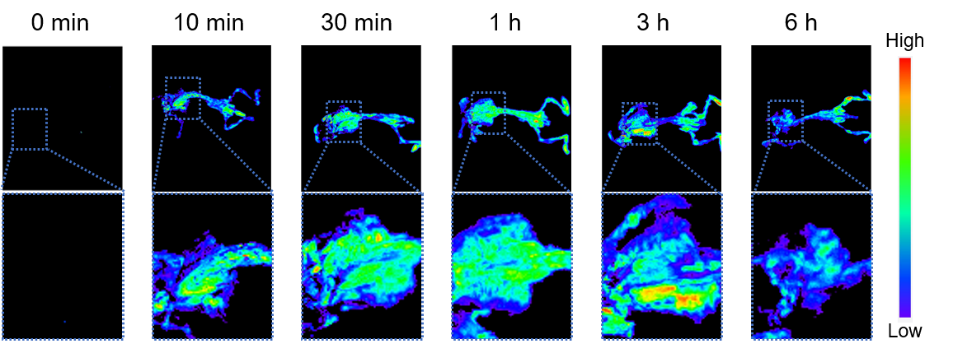


**Figure S9.** *In vivo* fluorescence images at diverse times post-injection of CMPA in mice.


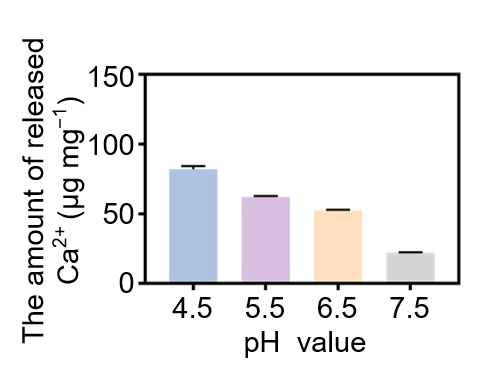


**Figure S10.** The total amount of Ca^2+^ released by CMPA at various pH values. Data are means ± s.d. (n ≥ 3).


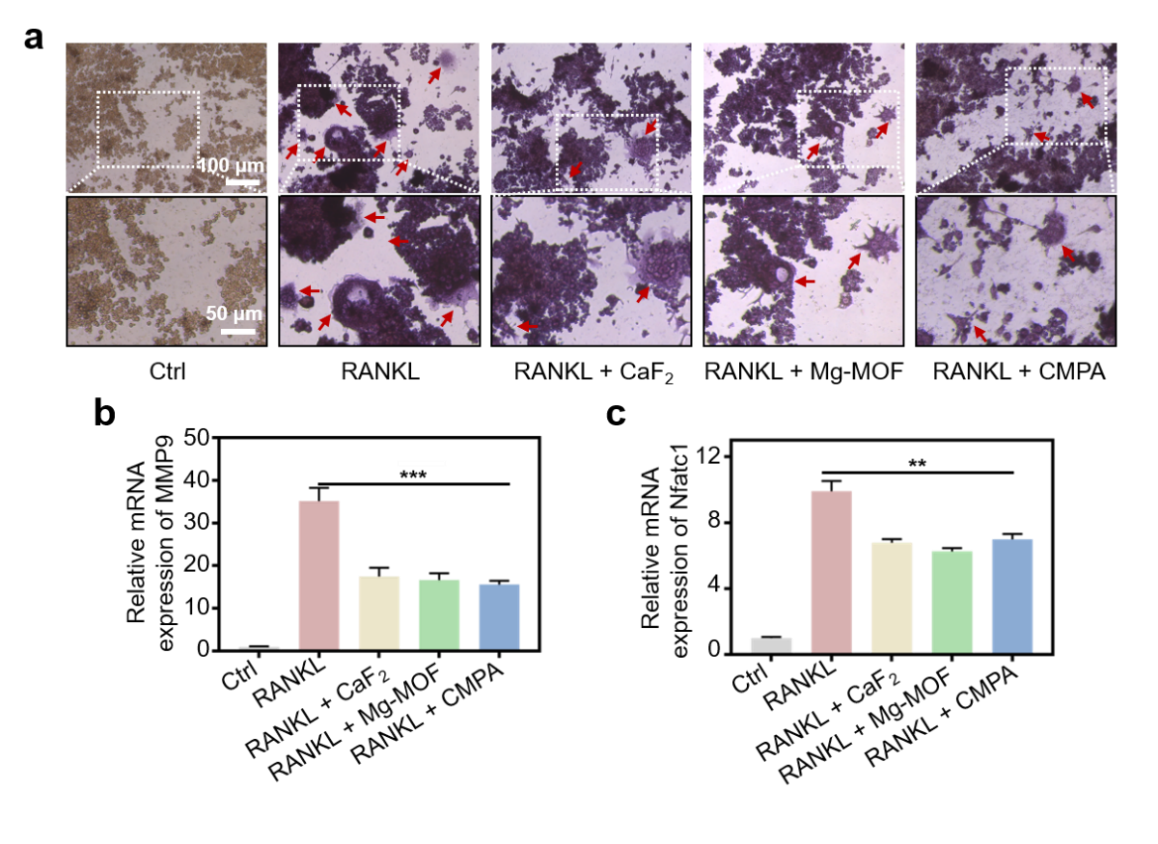


**Figure S11.** (a-c) TRAP staining images and the osteoclastogenic genes (Nfatc1 and MMP9) expressions in different groups on day 5. Red arrows represented osteoclasts. Data are means ± s.d. (n ≥ 3). **p* < 0.05, ***p* < 0.01, ****p* < 0.001.


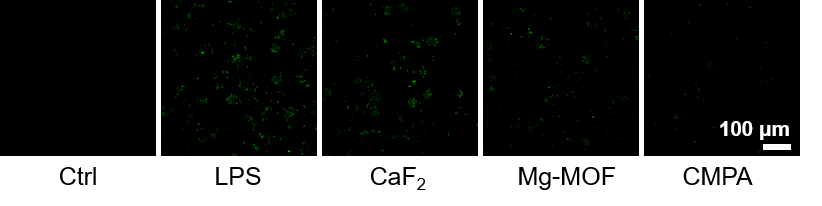


**Figure S12.** Fluorescence staining of ROS in RAW264.7 cells with varying treatments.


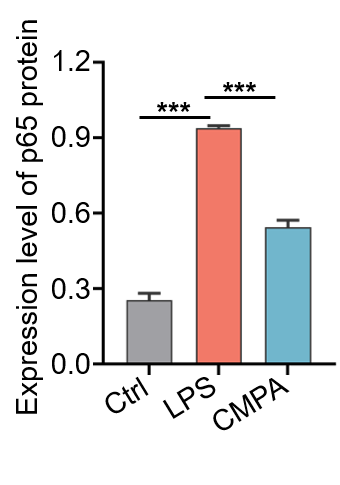


**Figure S13.** The expression level of p65 protein in various groups. Data are means ± s.d. (n ≥ 3). **p* < 0.05, ***p* < 0.01, ****p* < 0.001.


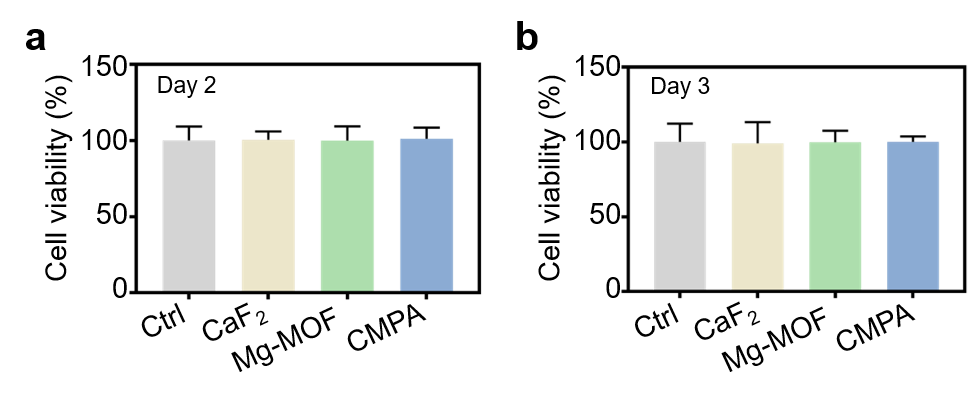


**Figure S14.** (a, b) The cytotoxicity of CaF_2_, Mg-MOF and CMPA on MC3T3-E1 cells for 2 and 3 days. Data are means ± s.d. (n ≥ 3).


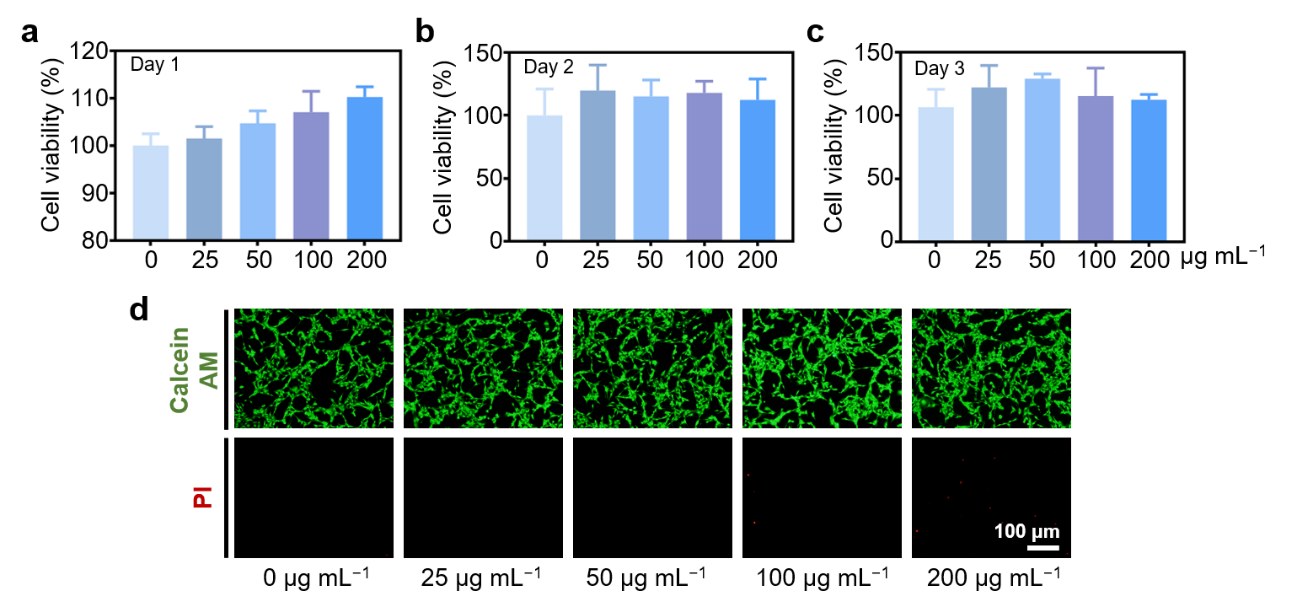


**Figure S15.** (a-c) The cell viability of MC3T3-E1 cells cocultured with different concentrations of CMPA for 1, 2 and 3 days. (d) Live/dead staining assay of MC3T3-E1 cells in diverse groups. Data are means ± s.d. (n ≥ 3).


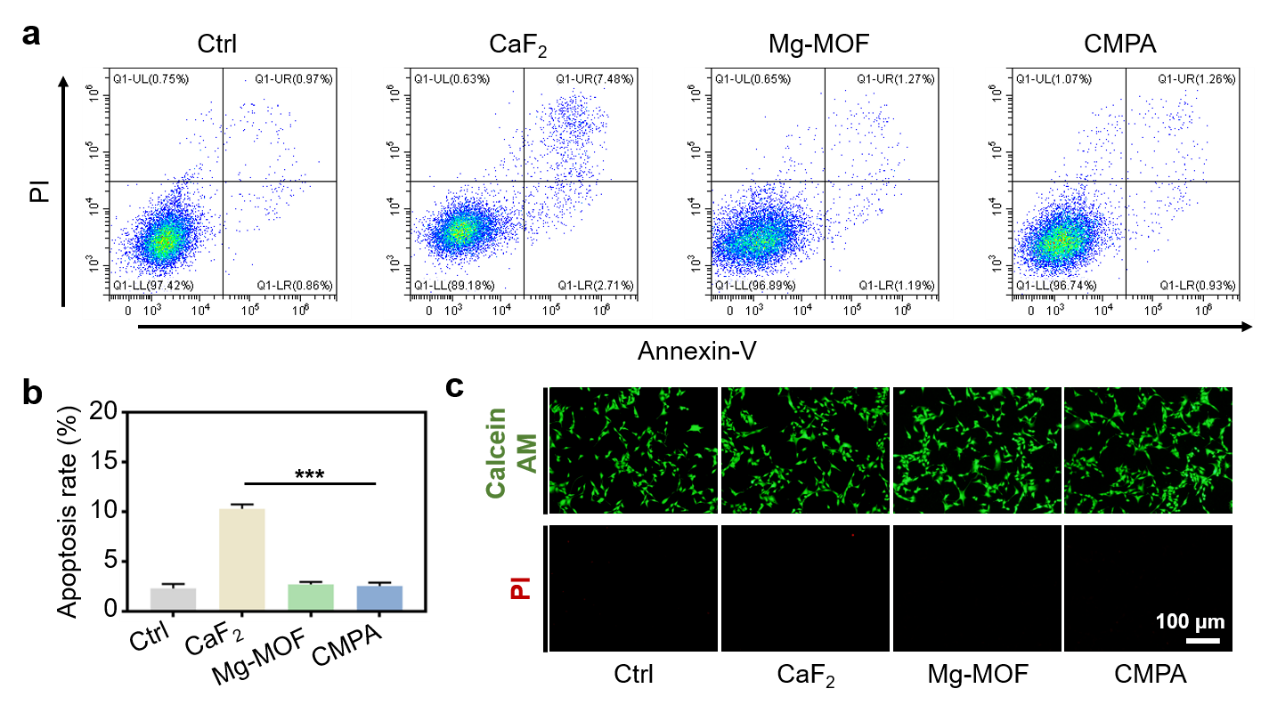


**Figure S16.** (a, b) Representative flow cytometric profiles and quantitative data of hMSCs cells by flow cytometry upon different treatments. (c) Live/dead staining results of hMSCs cells in various groups. Data are means ± s.d. (n ≥ 3). **p* < 0.05, ***p* < 0.01, ****p* < 0.001.


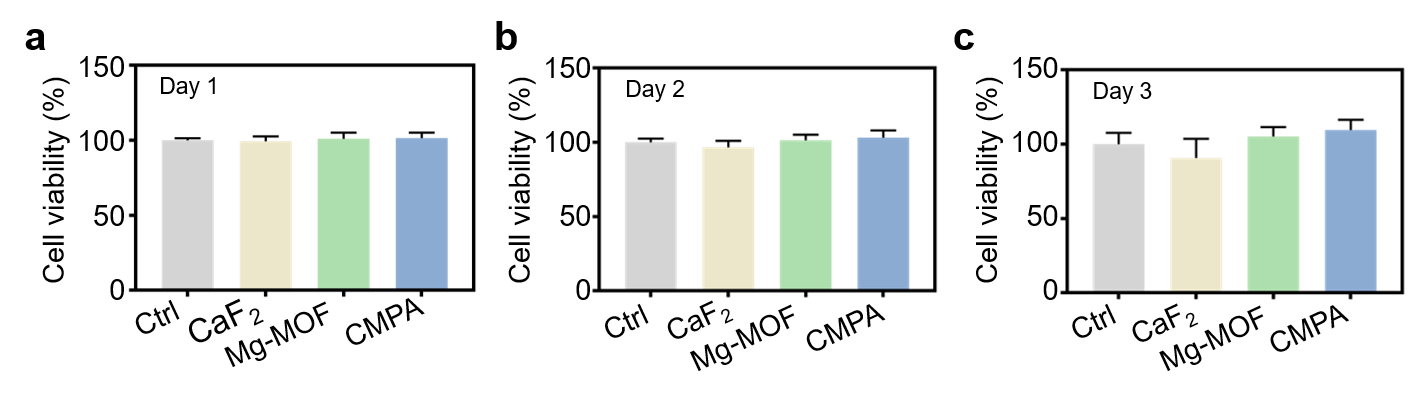


**Figure S17.** (a-c) The cytotoxicity of CaF_2_, Mg-MOF and CMPA on HUVECs cells for 1 day, 2 days and 3 days. Data are means ± s.d. (n ≥ 3).


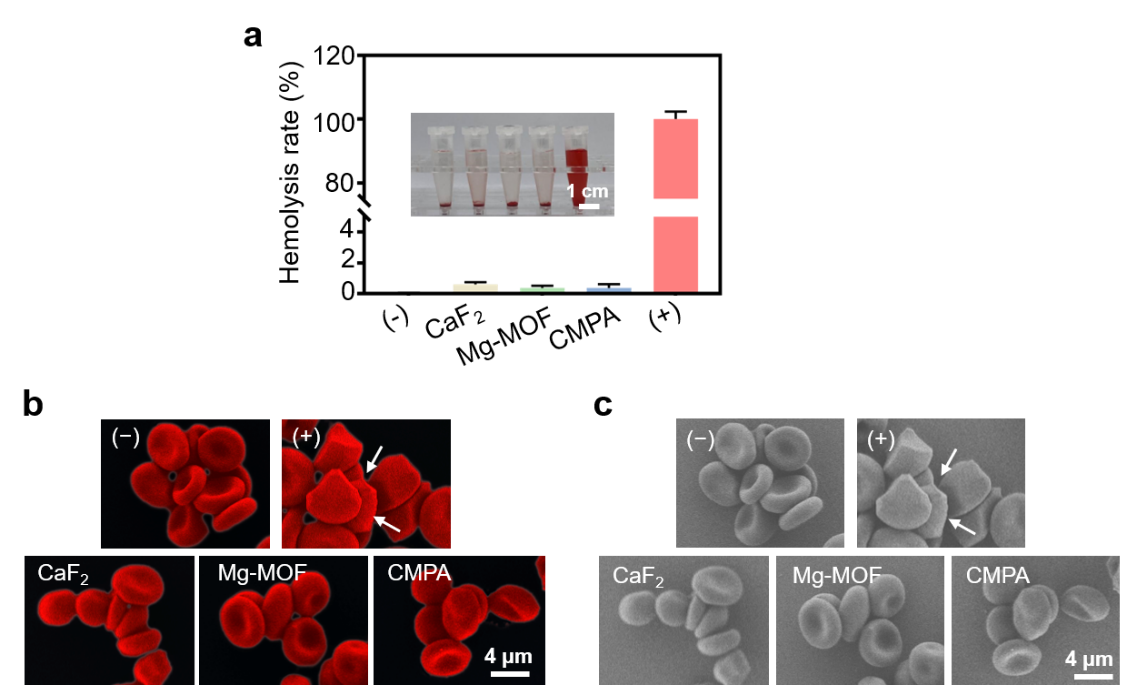


**Figure S18.** (a) Hemolysis analysis of RBCs upon different treatments. (b, c) SEM images of RBCs in various groups. Data are means ± s.d. (n ≥ 3).


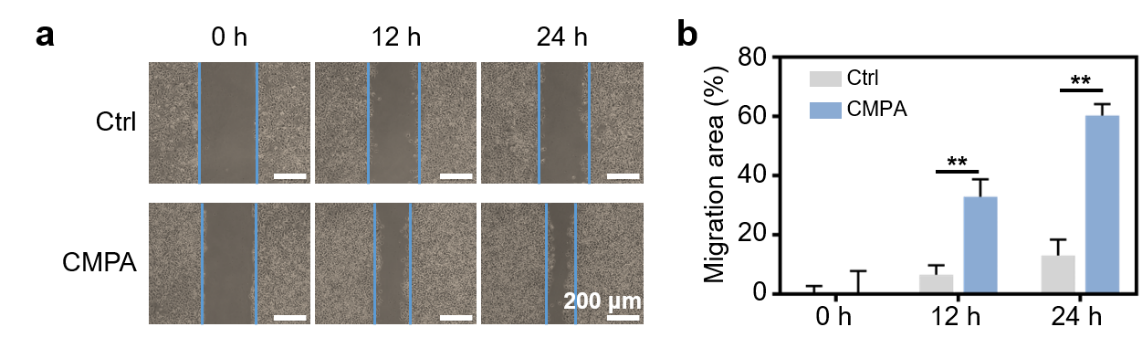


**Figure S19.** (a, b) Microscopic images and quantitative analysis of HUVECs cells scratch test in diverse groups. Data are means ± s.d. (n ≥ 3). **p* < 0.05, ***p* < 0.01, ****p* < 0.001.


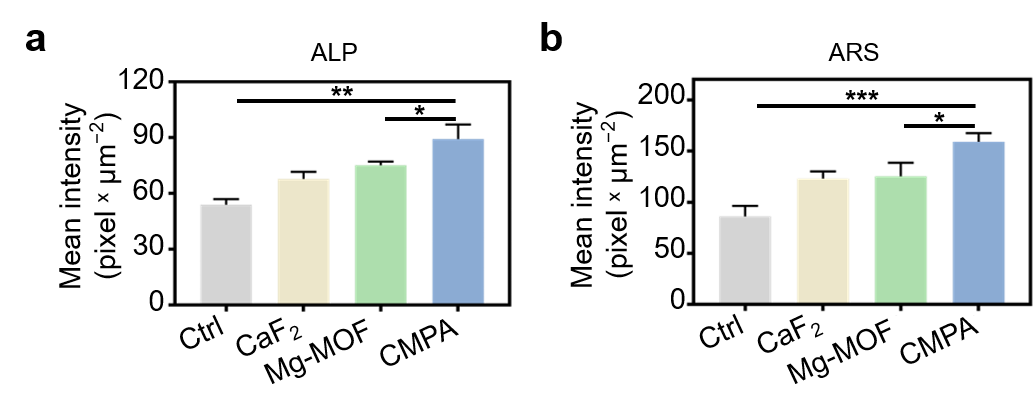


**Figure S20.** (a, b) Quantitative analysis of ALP and ARS staining images. Data are means ± s.d. (n ≥ 3). **p* < 0.05, ***p* < 0.01, ****p* < 0.001.


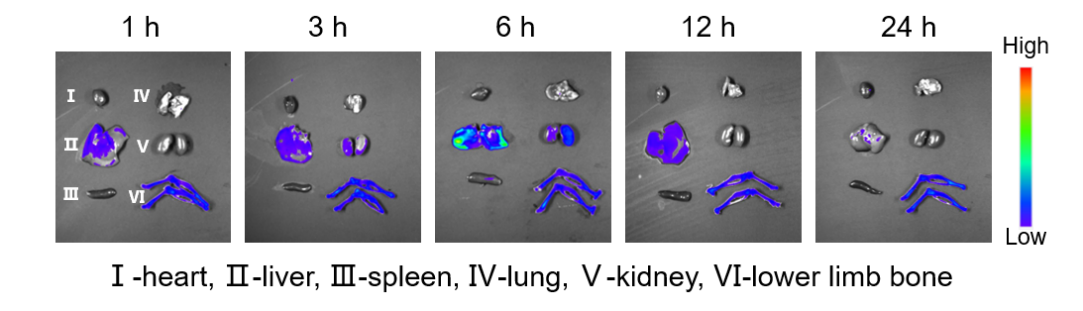


**Figure S21.** Representative fluorescence images of isolated organs and lower limb bones after injection with CMPA at different times.


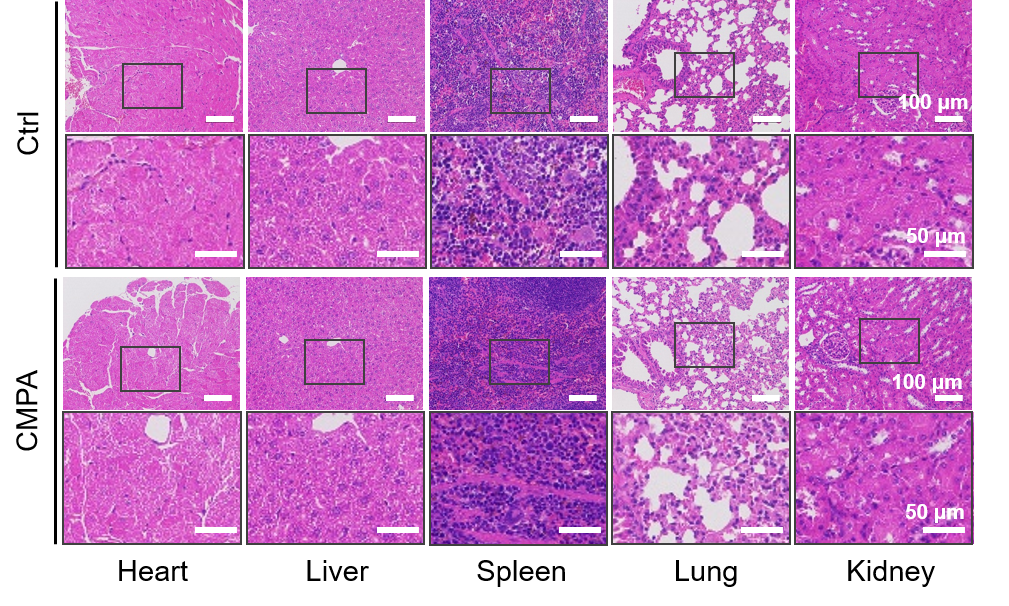


**Figure S22.** H&E staining of the heart, liver, spleen, lung and kidney of mice in various groups.


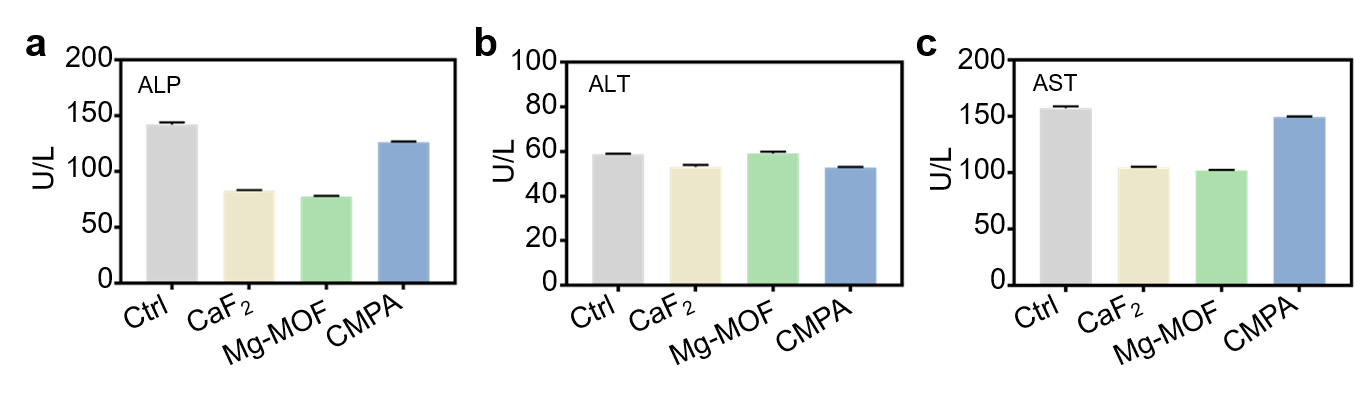


**Figure S23.** (a-c) Serum levels of ALP, ALT and AST of mice in diverse groups. Data are means ± s.d. (n ≥ 3).

**Table S1.** Quantitative-polymerase chain reaction primers utilized in this study.

| Gene | Forward primers (5’-3’) | Reverse primers (5’-3’) |
| --- | --- | --- |
| GAPDH | TGACTTCAACAGCGACACCCA | CACCCTGTTGCTGTAGCCAAA |
| OCN | ACCGAGACACCATGAGAGC | GCTGCACCTTTGCTGGA |
| RUNX2 | ACTCTACCACCCCGCTGTC | CAGAGGTGGCAGTGTCATCA |
| MMP9 | CTGGACAGCCAGACACTAAAG | CTCGCGGCAAGTCTTCAGAG |
| Nfatc1 | GAGAATCGAGATCACCTCCTAC | TTGCAGCTAGGAAGTACGTCTT |

**Table S2.** GO analysis referred to Figure 5d.

| ID | Term | *p*-value |
| --- | --- | --- |
| GO:0006955 | Immune response | 0.00000000002 |
| GO:0071222 | Cellular response to lipopolysaccharide | 0.00000000007 |
| GO:0006954 | Inflammatory response | 0.00000000985 |
| GO:0008285 | Negative regulation of cell population proliferation | 0.00000013956 |
| GO:0032760 | Positive regulation of tumor necrosis factor production | 0.00000087577 |
| GO:0010575 | Positive regulation of vascular endothelial growth factor production | 0.00000088413 |
| GO:0061469 | Regulation of type B pancreatic cell proliferation | 0.00000188113 |
| GO:0032743 | Positive regulation of interleukin-2 production | 0.00000473024 |
| GO:0032308 | Positive regulation of prostaglandin secretion | 0.00000489653 |
| GO:0046888 | Negative regulation of hormone secretion | 0.00000806836 |
| GO:0005615 | Extracellular space | 0.00000000225 |
| GO:0005576 | Extracellular region | 0.00000003477 |
| GO:0048786 | Presynaptic active zone | 0.00744943914 |
| GO:0048471 | Perinuclear region of cytoplasm | 0.01309169964 |
| GO:0043231 | Intracellular membrane-bounded organelle | 0.01325003547 |
| GO:0030526 | Granulocyte macrophage colony-stimulating factor receptor complex | 0.01431069269 |
| GO:0032059 | Bleb | 0.01714856977 |
| GO:0042585 | Germinal vesicle | 0.01714856977 |
| GO:0072536 | Interleukin-23 receptor complex | 0.01714856977 |
| GO:0098802 | Plasma membrane signaling receptor complex | 0.01714856977 |
| GO:0005125 | Cytokine activity | 0.00000000000 |
| GO:0008083 | Growth factor activity | 0.00006724633 |
| GO:0002020 | Protease binding | 0.00008892008 |
| GO:0035259 | Nuclear glucocorticoid receptor binding | 0.00165175154 |
| GO:0004364 | Glutathione transferase activity | 0.00359047282 |
| GO:0005164 | Tumor necrosis factor receptor binding | 0.00382277361 |
| GO:0004867 | Serine-type endopeptidase inhibitor activity | 0.00489913633 |
| GO:0030414 | Peptidase inhibitor activity | 0.00537070939 |
| GO:0019900 | Kinase binding | 0.00561638742 |
| GO:0008009 | Chemokine activity | 0.00621587022 |

**Table S3.** KEGG pathways enrichment analysis referred to Figure 5e.

| ID | Term | *p*-value |
| --- | --- | --- |
| mmu04060 | Cytokine-cytokine receptor interaction | 0.00000000001 |
| mmu04668 | TNF signaling pathway | 0.00000000001 |
| mmu04657 | IL-17 signaling pathway | 0.00000001868 |
| mmu04064 | NF-κB signaling pathway | 0.00000004887 |
| mmu05323 | Rheumatoid arthritis | 0.00000023134 |
| mmu04640 | Hematopoietic cell lineage | 0.00000669513 |
| mmu04625 | C-type lectin receptor signaling pathway | 0.00002080449 |
| mmu04630 | JAK-STAT signaling pathway | 0.00002329718 |
| mmu04061 | Viral protein interaction with cytokine and cytokine receptor | 0.00011160514 |
| mmu05321 | Inflammatory bowel disease | 0.00025680563 |
| mmu05133 | Pertussis | 0.00059691607 |
| mmu04933 | AGE-RAGE signaling pathway in diabetic complications | 0.00173033078 |
| mmu04659 | Th17 cell differentiation | 0.00192699028 |
| mmu04217 | Necroptosis | 0.00197736044 |
| mmu05146 | Amoebiasis | 0.00206640012 |
| mmu05152 | Tuberculosis | 0.00212938472 |
| mmu05144 | Malaria | 0.00276329783 |
| mmu05332 | Graft-versus-host disease | 0.00290928690 |
| mmu05134 | Legionellosis | 0.00354048461 |
| mmu04940 | Type I diabetes mellitus | 0.00406479169 |

**Table S4.** Routine blood test of mice in diverse groups.

| Index | Normal range | Ctrl | CMPA |
| --- | --- | --- | --- |
| RBC | 6.36-9.42 (10^12^/L) | 8.89 | 7.78 |
| WBC | 0.8-6.8 (10^9^/L) | 3.0 | 2.4 |
| PLT | 450-1590 (10^9^/L) | 878 | 762 |
| HGB | 110-143 (g/L) | 143 | 123 |
| HCT | 34.6-44.6 (%) | 44.6 | 37.6 |
| MCH | 15.8-19 (fL) | 16.1 | 15.8 |
| MCHC | 302-353 (pg) | 322 | 327 |
| MCV | 48.2-58.3 (fL) | 50.2 | 48.3 |

**Table S5.** List of abbreviations in order of appearance.

| Abbreviations | Full name |
| --- | --- |
| CMPA | CM-NH_2_-PAA-Ald |
| Mg^2+^ | Magnesium ions |
| Mg-MOF | Magnesium organic framework |
| Ca^2+^ | Calcium ions |
| CaF_2_ | Calcium-based upconversion nanoparticles |
| Ald | Alendronate sodium |
| OVX | Ovariectomy |
| CaCl_2_·6H_2_O | Calcium chloride hexahydrate |
| DHTA | 2,5-Dihydroxyterephthalic acid |
| DMF | N,N-dimethylformamide |
| Cit | Sodium citrate |
| NH_4_F | Ammonium fluoride |
| NaOH | Sodium hydroxide |
| Yb(NO_3_)_3_·5H_2_O | Ytterbium nitrate pentahydrate |
| Er(NO_3_)_3_·6H_2_O | Erbium nitrate hexahydrate |
| Mg(NO_3_)_2_·6H_2_O | Magnesium nitrate hexahydrate |
| HAP | Hydroxyapatite |
| CCK-8 | Cell counting kit-8 |
| DCFH-DA | 2’,7’-Dichlorofluorescein diacetate |
| TRAP | Ttartrate resistant acid phosphatase |
| ALP | Stain kit and alkaline phosphatase |
| ARS | Alizarin red S |
| Calcein-AM | Calcein-acetoxymethyl ester |
| PI | Propidium iodide |
| FBS | Fetal bovine serum |
| DMEM | Dulbecco’s modified eagle culture medium |
| TEM | Transmission electron microscopy |
| SEM | Scanning electron microscope |
| XRD | X-ray diffraction |
| XPS | X-ray photoelectron spectroscopy |
| UV-vis | Ultraviolet-visible |
| FTIR | Fourier transform infrared spectroscopy |
| FITC | Fluorescein 5-isothiocyanate |
| PBS | Phosphate buffer solution |
| ICP | Inductively coupled plasma |
| RANKL | Receptor activator of nuclear factor kappa-B ligand |
| qRT-PCR | Quantitative real-time polymerase chain reaction |
| MMP9 | Matrix metalloproteinase 9 |
| Nfatc1 | Nuclear factor of activated T cells |
| HUVECs | Human umbilical vein endothelial cells |
| hMSCs | Human bone marrow mesenchymal stem cells |
| MC3T3-E1 | Mouse pre-osteoblast cells |
| OD | Optical density |
| ROS | Reactive oxygen species |
| LPS | Lipopolysaccharide |
| IL-6 | Interleukin-6 |
| TNF-α | Tumor necrosis factor-α |
| IL-10 | Interleukin-10 |
| TGF-β | Transforming growth factor-β |
| ELISA | Enzyme-linked immunosorbent assay |
| DAPI | 4’,6-Diamidino-2-phenylindole |
| WB | Western blot |
| RNA-seq | Transcriptome sequencing |
| RBCs | Red blood cells |
| RUNX2 | Runt-related transcription factor 2 |
| OCN | Osteocalcin |
| micro-CT | Micro-computed tomography |
| BMD | Bone mineral density |
| BV/TV | Bone volume per tissue volume |
| Tb.N | Trabecular number |
| Tb.Sp | Trabecular separation |
| Tb.Th | Trabecular thickness |
| 3D | Three-dimensional |
| H&E | Hematoxylin and eosin |
| Ctrl | Control |
| ALT | Alanine transaminase |
| AST | Aspartate transaminase |
| PCA | Principal component analysis |
| DEGs | Differentially expressed genes |
| IL-1α | Interleukin-1α |
| IL-1β | Interleukin-1β |
| GO | Gene ontology |
| KEGG | Kyoto encyclopedia of genes and genomes |
| TNF | Tumor necrosis factor |
| IL-17 | Interleukin-17 |
| PPI | Protein-protein interactions |
| GSEA | Gene set enrichment analysis |
| p-p65 | Phospho-p65 |
